# A single-dose MCMV-based vaccine elicits long-lasting immune protection in mice against distinct SARS-CoV-2 variants

**DOI:** 10.3389/fimmu.2024.1383086

**Published:** 2024-07-25

**Authors:** Kristin Metzdorf, Henning Jacobsen, Yeonsu Kim, Luiz Gustavo Teixeira Alves, Upasana Kulkarni, Maja Cokarić Brdovčak, Jelena Materljan, Kathrin Eschke, M. Zeeshan Chaudhry, Markus Hoffmann, Federico Bertoglio, Maximilian Ruschig, Michael Hust, Marko Šustić, Astrid Krmpotić, Stipan Jonjić, Marek Widera, Sandra Ciesek, Stefan Pöhlmann, Markus Landthaler, Luka Čičin-Šain

**Affiliations:** ^1^ Department of Viral Immunology, Helmholtz Centre for Infection Research, Braunschweig, Germany; ^2^ Centre for Individualized Infection Medicine, a Joint Venture of the Helmholtz Centre for Infection Medicine and the Hannover Medical School, Hannover, Germany; ^3^ Berlin Institute for Medical Systems Biology (BIMSB), Max Delbrück Center for Molecular Medicine in the Helmholtz Association (MDC), Berlin, Germany; ^4^ Center for Proteomics, University of Rijeka, Faculty of Medicine, Rijeka, Croatia; ^5^ Department of Histology and Embryology, University of Rijeka, Faculty of Medicine, Rijeka, Croatia; ^6^ Infection Biology Unit, German Primate Center – Leibniz Institute for Primate Research, Göttingen, Germany; ^7^ Faculty of Biology and Psychology, Georg-August-University Göttingen, Göttingen, Germany; ^8^ Department of Medical Biotechnology, Institute for Biochemistry, Biotechnology and Bioinformatics, Technische Universität Braunschweig, Braunschweig, Germany; ^9^ Institute for Medical Virology, University Hospital Frankfurt, Goethe University Frankfurt, Frankfurt am Main, Germany; ^10^ Fraunhofer Institute for Translational Medicine and Pharmacology ITMP, Frankfurt am Main, Germany; ^11^ German Centre for Infection Research (DZIF), External Partner Site Frankfurt, Frankfurt, Germany; ^12^ Institute for Biology, Humboldt-Universität zu Berlin, Berlin, Germany

**Keywords:** SARS-CoV-2, COVID-19, MCMV, vaccination, single-dose, long-lasting protection, mouse, *in vivo*

## Abstract

Current vaccines against COVID-19 elicit immune responses that are overall strong but wane rapidly. As a consequence, the necessary booster shots have contributed to vaccine fatigue. Hence, vaccines that would provide lasting protection against COVID-19 are needed, but are still unavailable. Cytomegaloviruses (CMVs) elicit lasting and uniquely strong immune responses. Used as vaccine vectors, they may be attractive tools that obviate the need for boosters. Therefore, we tested the murine CMV (MCMV) as a vaccine vector against COVID-19 in relevant preclinical models of immunization and challenge. We have previously developed a recombinant MCMV vaccine vector expressing the spike protein of the ancestral SARS-CoV-2 (MCMV^S^). In this study, we show that the MCMV^S^ elicits a robust and lasting protection in young and aged mice. Notably, spike-specific humoral and cellular immunity was not only maintained but also even increased over a period of at least 6 months. During that time, antibody avidity continuously increased and expanded in breadth, resulting in neutralization of genetically distant variants, like Omicron BA.1. A single dose of MCMV^S^ conferred rapid virus clearance upon challenge. Moreover, MCMV^S^ vaccination controlled two variants of concern (VOCs), the Beta (B.1.135) and the Omicron (BA.1) variants. Thus, CMV vectors provide unique advantages over other vaccine technologies, eliciting broadly reactive and long-lasting immune responses against COVID-19.

## Introduction

Coronavirus disease 2019 (COVID-19), caused by the severe acute respiratory syndrome coronavirus 2 (SARS-CoV-2) (1), had a massive impact on public health. The virus rapidly spread across multiple countries in 2020, leading to millions of confirmed cases and deaths in the subsequent years (2). The elderly have been particularly affected by SARS-CoV-2, with a higher risk of severe COVID-19. Early during the pandemic, SARS-CoV-2 only exhibited little genetic variation, with only a single spike substitution (D614G) in the PANGO lineage B.1, which rapidly became dominant in Europe in late 2020 (3). However, a large number of new viral variants emerged in 2021, characterized by an increasing number of mutations in the spike (S) protein sequence and immune escape potential, including the Beta (B.1.351) (4), the Delta (B.1.617.2) (5), or the Omicron variant (B.1.1.529) (6) and its sub-variants. Numerous COVID-19 vaccines that induce immune responses against the spike glycoprotein have been developed and authorized for use with unprecedented speed. However, vaccine-induced immunity wanes within months, resulting overall in reduced protection against infections and disease (4, 7–9). In addition, emerging variants have been increasingly capable of escaping vaccine-induced immunity, driving the need for continued vaccine adaptation and booster shots (10–12). Thus, acute (13) or long-term (14–16) effects after SARS-CoV-2 infection remain a public health issue, and a vaccine that confers lasting and broad immune protection remains unmet.

Recently, our research group has demonstrated that a mouse cytomegalovirus (MCMV)-based COVID-19 vaccine candidate, encoding the spike protein derived from the Index (Wuhan) isolate (MCMV^S^), elicits robust and persistent humoral as well as cellular immune responses in mice (17). MCMV is a well-characterized model virus for human CMV (HCMV) infection (18). CMVs are strictly species-specific for their cognate host and induce robust anti-viral immunity, which persists over a lifetime (19–22). This long-term maintenance of antigen-specific lymphocytes, known as memory inflation (22, 23), is characterized by an accumulation and persistence of CD8^+^ T cells with an effector memory (T_EM_) phenotype, recognizing immuno-dominant viral antigens (23, 24). Therefore, transgenic antigens from other infectious agents have been expressed in recombinant MCMV (25), and such vaccine vectors were shown to provide long-term immune protection (26). This concept was validated in other models of CMV immunization, where a peculiarly strong CD8^+^ T-cell immunity was crucial for immune protection (26–31). More recently, it was noticed that MCMV infection also results in lasting humoral immune responses toward viral antigens (32), which was exploited to retarget immune protection against other viruses (33), including influenza A virus (17). The spike protein of the Index strain of SARS-CoV-2 does not bind to murine ACE2 (mACE2) receptors. Hence, mouse models of SARS-CoV-2 infection were initially restricted to transgenic mice expressing the human ACE2 receptor (hACE2), or mouse-adapted SARS-CoV-2 variants. However, the naturally occurring N501Y mutation in the spike protein is sufficient for mACE2 binding (34). Since this mutation was present in various variants of concern (VOCs), including Beta and Omicron BA.1, these variants could also be used in infection experiments with non-transgenic mice once they emerged (35).

Here, we show that a single-dose immunization with MCMV^S^ elicited virus neutralizing titers (VNTs) that increased for at least 6 months, concomitant with an increase of antibody avidity and resulting in a broadening of neutralization effects to genetically distant variants. The immunization protected vaccinated K18-hACE2 mice against mortality and disease upon challenge with SARS-CoV-2 D614G or the Delta variant. Furthermore, the same vaccination protected BALB/c mice against challenge with the SARS-CoV-2 Beta and Omicron BA.1 variants, and the protection lasted for at least 5 months. Hence, our results argue that MCMV-based COVID-19 vaccines may provide long-term and broad protection against multiple SARS-CoV-2 VOCs.

## Results

### Protection against SARS-CoV-2 D614G-mediated disease elicited by a single dose of MCMV^S^-vaccine

**Figure 1:**
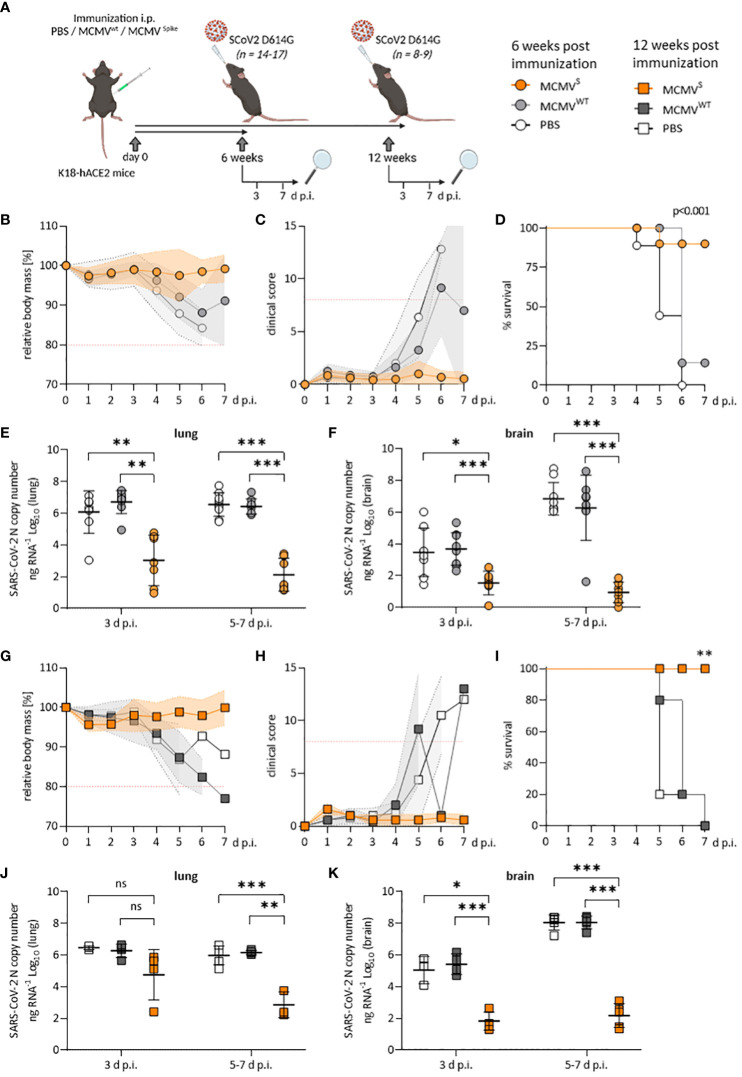
Protection against SARS-CoV-2 D614G-mediated disease by a single dose of MCMV^S^-vaccine. **(A)** Schematic representation of the experimental setup. Mice were challenged with 2x10^3^ PFU of SARS-CoV-2 D614G at six weeks **(B–F)** or twelve weeks **(G–K)** post-immunization. Created with BioRender.com. **(B)** Relative body mass, **(C)** clinical scoring, and **(D)** survival of SARS-CoV-2 D614G challenged mice, six weeks after treatment/immunization with PBS (n=9), MCMVWT (n=8), or MCMV^S^ (n=14). **(E, F)** Viral loads as SARS-CoV-2 N gene copy numbers per ng RNA at 3 d p.i. (PBS n=8, MCMV^WT^ n=9, MCMVS n=7) or 5-7 d p.i. (PBS n=9, MCMV^W T^n=8, MCMV^S^ n=7) in lung **(E)** or brain homogenates **(F)**. Seven MCMV^S^-immunized mice were followed up until 21 days post-challenge and were only included for clinical analysis **(B–D)** as their RNA-loads were undetectable (data not shown). Animals that were sacrificed at 3 d p.i. were not included in clinical analyses. **(G)** Relative body mass, **(H)** clinical scoring, and **(I)** survival of SARS-CoV-2 D614G challenged mice, twelve weeks after treatment/immunization with PBS (n=5), MCMV^WT^ (n=5), or MCMV^S^ (n=5). **(J, K)** Viral loads as SARS-CoV-2 N gene copy numbers per ng RNA at 3 d p.i. (PBS n=3, MCMV^WT^ n=4, MCMV^S^ n=5) or 5-7 d p.i. (PBS n=5, MCMV^W T^n=5, MCMV^S^ n=5) in lung **(J)** or brain homogenates **(K)**. Animals that were sacrificed at 3 d p.i. were not included in clinical analyses. All data represent pooled data from two independent experiments. All data (except in D, and I) are shown as mean ± SD. Red-dotted lines in **(B, C, G, H)** indicate the maximal acceptable burden for animal experiments. The humane endpoint (>20% reduction in body mass **(B, G)** or a clinical score of ≥8 **(C, H)**) was pre-defined by authorized animal trial permits. For survival analyses **(D, I)**, a log-rank (Mantel-cox) test was used to assess statistical significance. Statistical significance for longitudinal assessments **(B, C, G, H)** was calculated with Greenhouse-Geisser corrected Two-Way ANOVA and Tukey post-hoc testing. All other comparisons **(E, F, J, K)** were statistically assessed using Brown-Forsythe and One-Way ANOVA and Dunnett T3 correction for multiple comparisons (two-tailed) (* *p* < 0.05, ** *p* <0.01, *** *p* < 0.001, ns = not significant).

We have previously shown that a single dose of an MCMV-based vaccine vector encoding the spike antigen (MCMV^S^) from SARS-CoV-2 triggers a robust inflationary CD8^+^ T-cell response against the spike-encoded octameric peptide VNFNFNGL in C57BL/6 mice as well as neutralizing antibody responses that persisted in BALB/c mice for a minimum of 90 days (17). In this study, we immunized K18-hACE2 mice, which express the human ACE2 receptor (36) and thus may be infected by early variants of SARS-CoV-2. We used MCMV^S^, a vector control (MCMV^WT^), or PBS for immunization/mock treatment and challenged the mice with a potentially lethal dose of SARS-CoV-2 D614G, 6 or 12 weeks later (**Figure 1A**).

To quantify the CD8^+^ T-cell response, blood was sampled before SARS-CoV-2 challenge, and blood leukocytes were analyzed by flow cytometry (gating strategy shown in **Supplementary Figure 1**). MCMV^S^ and MCMV^WT^ elicited comparable frequencies of CD8^+^ T cells in total, primed (CD44^+^CD11a^+^), effector (CD62L^-^, T_EFF_), terminally differentiated effector (CD62L^-^, KLRG1^+^, T_TDE_), effector memory (CD62L^-^, KLRG1^-^, T_EM_), and central memory (CD62L^+^, T_CM_) compartments (**Supplementary Figure 2B**). However, only MCMV^S^-vaccinated mice displayed high frequencies of SARS-CoV-2 spike peptide (VNFNFNGL)-specific CD8^+^ T cells (**Supplementary Figure 2C**). The frequency of these cells was approximately twofold increased at 3 months over 1 month post-immunization in all tested subsets (**Supplementary Figure 2C**), and this increase over time was confirmed by dynamic monitoring of VNFNFNGL-specific CD8^+^ T cells in the blood (**Supplementary Figure 2D**). Likewise, neutralizing titers were typically higher at 3 months than at 1 month post-immunization (**Supplementary Figure 2E**), in line with our previous report in non-transgenic mice (17).

We challenged these mice with 2 × 10^3^ PFU of SARS-CoV-2 D614G at the indicated time points (**Figure 1A**) and monitored body mass and clinical scores to evaluate protection against disease. Immunized animals that robustly responded to vaccination (**Supplementary Figure 2**) were protected against disease at 6 weeks, as evidenced by maintained body mass (**Figure 1B**) and by the absence of clinical signs (**Figure 1C**), while most mock-vaccinated mice became severely ill, reaching the humane endpoint by day 5 post-infection (d p.i.) or earlier (**Figure 1D**). At 3 and at 5–7 d p.i., MCMV^S^-vaccination decreased the median SARS-CoV-2 viral RNA load in lungs and brains by a factor of 1,000 or more relative to mock-vaccinated groups (**Figures 1E, F**). The results were similar in the trachea, stomach, heart, and spleen (**Supplementary Figure 3A**). The immunity of clinically approved vaccines against COVID-19 gradually wanes over time (37, 38). To assess if the protective effects of MCMV^S^ are reduced over time, mice were challenged at 3 months post-immunization. All MCMV^S^-vaccinated mice were protected against disease, in terms of body mass reduction (**Figure 1G**), clinical signs (**Figure 1H**), and survival (**Figure 1I**). SARS-CoV-2 RNA loads were reduced in the MCMV^S^-immunized group in all examined organs (**Figures 1J, K** and **Supplementary Figure 3B**). Some MCMV^S^-vaccinated mice presented viral RNA in lungs at 3 d p.i., but only minor amounts of viral RNAs were detected in this organ at 7 d p.i. (**Figure 1J**), arguing that a single administration of the MCMV^S^ vaccine inhibits virus replication. Moreover, the viral RNA load in the brain was >10,000-fold higher in the controls compared to the MCMV^S^-immunized mice at days 5 to 7 post-challenge (**Figure 1K**). In sum, a single-dose MCMV^S^ immunization protected animals from SARS-CoV-2-mediated disease at 6 and 12 weeks post-vaccination.

### Long-lived neutralizing antibody responses against SARS-CoV-2 Beta and Omicron variants upon MCMV^S^ immunization

**Figure 2:**
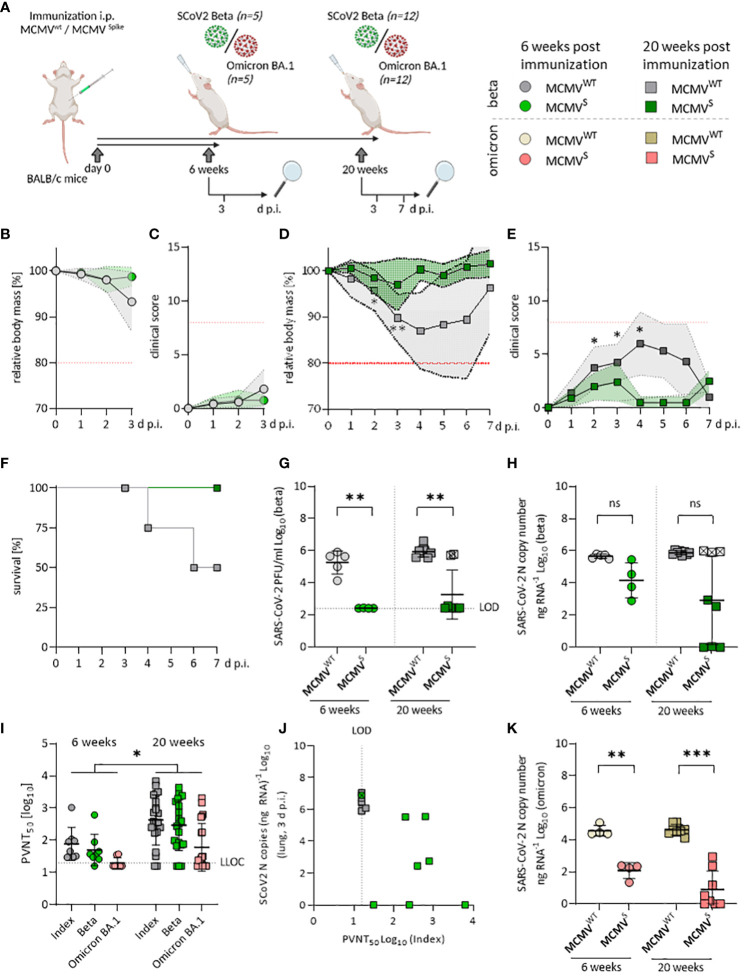
Sustained neutralizing antibody responses against SARS-CoV-2 Beta and Omicron BA.1 upon MCMV^S^-vaccination. **(A)** Schematic representation of the experimental setup. Mice were challenged with 6x10^4^ PFU of SARS-CoV-2 in all settings. Created with BioRender.com. **(B)** Relative body mass, and **(C)** clinical scoring of SARS-CoV-2 Beta challenged mice, six weeks after treatment/immunization with MCMV^WT^ (n=5), or MCMV^S^ (n=4). **(D)** Relative body mass, **(E)** clinical scoring, and **(F)** survival of SARS-CoV-2 Beta challenged mice, 20 weeks after treatment/immunization with MCMV^WT^ [n=12 (until day 3 n=8, day 7 n=4)], or MCMV^S^ [n=12 (until day 3 n=8, day 7 n=4)]. **(G)** Infectious virus titers, and **(H)** Viral loads in murine lungs three days after challenge with SARS-CoV‑2 Beta at six weeks (MCMV^WT^ n=4, MCMV^S^ n=5), and 20 weeks (MCMV^WT^ n=8, MCMV^S^ n=8) post-immunization. **(I)** Pseudo-virus neutralization serum titers (PVNT_50_) against SARS-CoV-2 Index, Beta, or Omicron BA.1 in mice at six weeks (MCMV^S^ n=8), and 20 weeks (MCMVS n=21) post-immunization with MCMVS (pre-challenge). **(J)** Correlation of viral loads in lungs and PVNT_50_ against the Index variant of SARS-CoV-2 Beta-challenged mice, 20 weeks post-immunization. One MCMV^S^-immunized mouse that did not show any neutralization titer is marked with a crossed symbol. **(K)** Viral loads in murine lungs three days after challenge with SARS-CoV‑2 Omicron BA.1 at six weeks (MCMV^WT^ n=4, MCMV^S^ n=8), and 20 weeks (MCMV^WT^ n=4, MCMV^S^ n=8) post-immunization. All data (except in **F**, and **J**) are shown as mean ± SD. Black dotted lines indicate the limit of detection (LOD) or lower limit of confidence (LLOC). Red dotted lines in **(B–E)** indicate the maximal acceptable burden for animal experiments. The humane endpoint (>20% reduction in body mass **(B, D)** or a clinical score of ≥8 **(C, E)**) was pre-defined by authorized animal trial permits. For survival analyses **(F)**, a log-rank (Mantel-cox) test was used to assess statistical significance. Statistical significance for longitudinal assessments **(B–E)** was calculated with Greenhouse-Geisser corrected Two-Way ANOVA and Tukey post-hoc testing. All other comparisons **(G–I, K)** were statistically assessed using Brown-Forsythe and One-Way ANOVA and Dunnett T3 correction for multiple comparisons (two-tailed) (* p < 0.05, ** p <0.01, *** p < 0.001, ns = not significant).

We next tested if MCMV^S^ elicited immunity against the SARS-CoV-2 Beta (B.1.351) and Omicron (BA.1) variants. Since both of these variants contain the N501Y mutation in the spike protein, infection with these variants is possible in non-transgenic mice, such as BALB/c and C57BL/6 (34). The spike used in MCMV^S^ corresponds to the Index variant of SARS-CoV-2, and therefore does not engage the mACE2 receptor in such mice, potentially resulting in fewer antigen-specific side effects. To assess whether MCMV^S^ can protect BALB/c in the long term, we challenged mice at 20 weeks post-vaccination (referred to as long-term cohort) with the SARS-CoV-2 Beta or Omicron BA.1 variants. We also challenged a cohort at 6 weeks after immunization (referred to as short-term cohort) with a 3-day follow-up after challenge (**Figure 2A**). In the short-term cohort, MCMV^S^-immunized animals retained their body mass shortly after infection and displayed only minimal clinical symptoms, while MCMV^WT^ mock-immunized animals lost approximately 10% of their initial body mass at day 3 post-infection (**Figures 2B, C**).

In the long-term cohort, MCMV^S^-vaccinated animals remained protected against clinical disease upon challenge with the Beta variant when followed for 7 days post-infection (**Figures 2D, E**). Two out of four animals (50%) in the mock-immunized group that were followed up until day 7 post-challenge reached the humane endpoint, while this occurred in none of the immunized animals (**Figure 2F**). Challenge with SARS-CoV-2 Omicron BA.1 resulted in no discernible body mass reduction in any of the short-term groups (**Supplementary Figure 4A**) and long-term groups (**Supplementary Figure 4B**). Altogether, our data indicate that a single dose of MCMV^S^ vaccination provided a robust and lasting protection against the Beta variant of SARS-CoV-2, while protection against clinical symptoms induced by Omicron BA.1 infection was hard to assess due to overall low pathogenicity of this variant in mice.

Vaccine efficacy was further assessed by measuring SARS-CoV-2 RNA copies and infectious titers in the lungs at 3 days post-infection. Both short- and long-term MCMV^S^-immunized animals harbored significantly fewer infectious particles in the lungs than the control groups (**Figure 2G**). However, three immunized animals from the long-term cohort showed high viral RNA loads upon infection with the Beta variant and two of the samples also had high viral titers (**Figures 2G, H**). In clinical settings, protection against infection and SARS-CoV-2-mediated disease is associated with the presence of neutralizing antibodies (nAbs) targeting SARS-CoV-2 (39, 40). Hence, we tested the neutralizing capacity of antibodies targeting different variants of SARS-CoV-2 before (**Figure 2I**) and after SARS-CoV-2 Beta (**Supplementary Figure 4C**) or Omicron BA.1 challenge (**Supplementary Figure 4D**). The nAb titers against the SARS-CoV-2 Index, Beta, and Omicron BA.1 variants were significantly higher at 20 weeks than at 6 weeks post-immunization (**Figure 2I**). Pseudo-virus neutralization titers correlated to decreased viral loads in the lungs and, of note, two MCMV^S^-immunized animals showed very low neutralization titers (**Figure 2J**), indicating that immunization of these animals failed. Importantly, MCMV^S^-vaccinated animals showed a strong capacity to control replication of the SARS-CoV-2 Omicron BA.1 variant. We observed significant, >100-fold reduced viral RNA loads in the short-term cohort and >10,000-fold reduced virus loads in the long-term challenge scenario (**Figure 2K**). Infectious Omicron BA.1 virus remained undetectable in any scenario (data not shown).

Collectively, a receptor-inert SARS-CoV-2 Index spike antigen delivered through an MCMV-based vector prompted robust and persistent nAb reactions, with no signs of immune waning and predictively correlated with a decrease in SARS-CoV-2 viral loads upon challenge.

### A single-dose immunization with MCMV^S^ provides inflationary immunogenicity in aged mice

**Figure 3:**
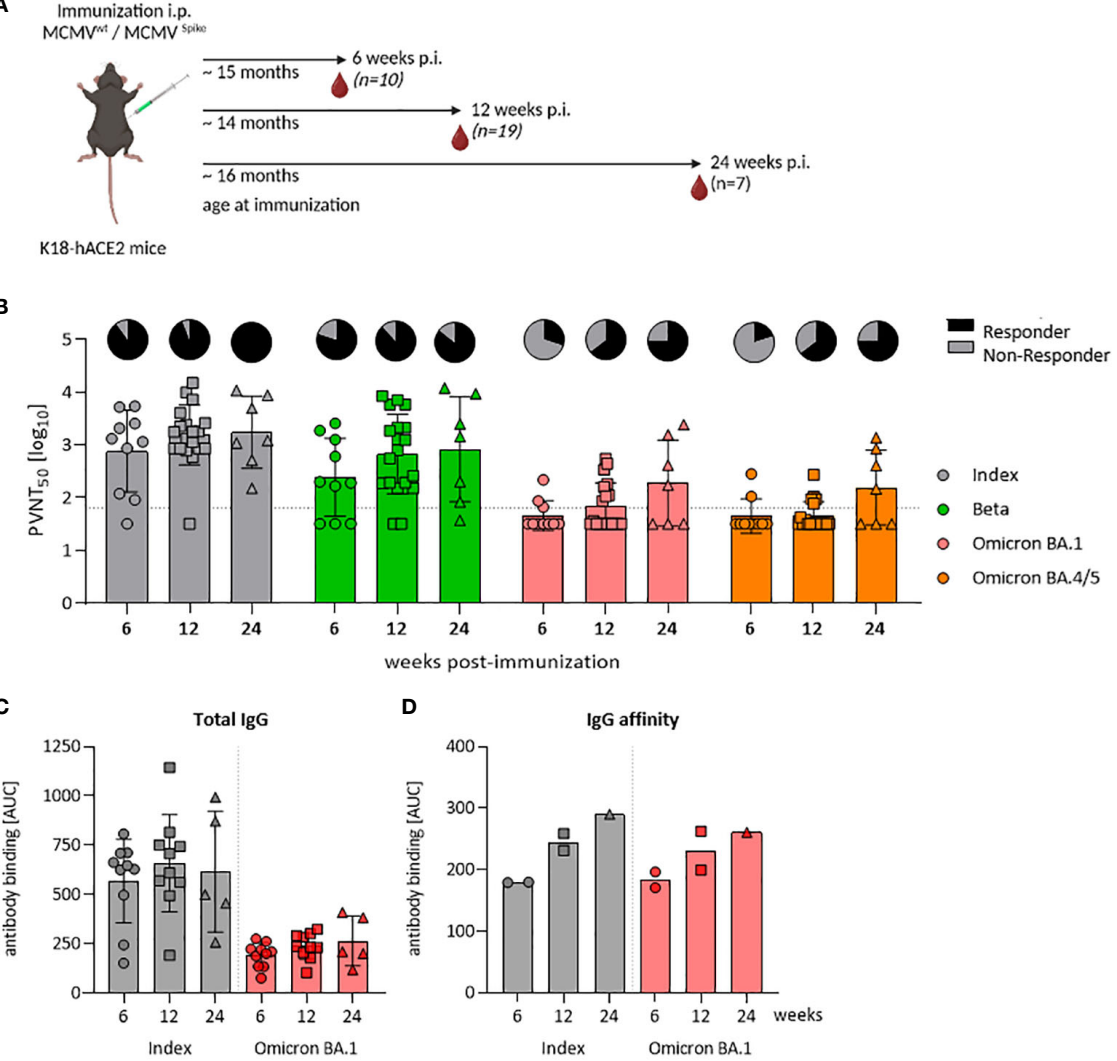
A single-dose of MCMV^S^ provides inflationary immunogenicity in aged mice. **(A)** Schematic representation of the experimental setup. Created with BioRender.com. **(B)** Pseudo-virus neutralization serum titers (PVNT_50_) against SARS-CoV-2 Index, Beta, Omicron BA.1, or Omicron BA.4/5 in mice at six (MCMV^S^ n=10), twelve (MCMV^S^ n=19), or 24 weeks (MCMV^S^ n=7) post-immunization with MCMVS. Pie charts indicate the percentage [%] of animals, with titers above the lower limit of confidence (responder versus non-responder). **(C)** SARS-CoV-2 spike-specific total IgG response by ELISA against the Index and Omicron BA.1 variants in murine sera at six (MCMVS n=10), twelve (MCMV^S^ n=10), and 24 (MCMV^S^ n=5) weeks post-immunization. **(D)** IgG affinity against SARS-CoV-2 Index and Omicron BA.1 spike measured by antibody binding [AUC] in presence of graded amounts of NaSCN using ELISA. Each data point in **(D)** represents a pool of three mice from the same group. All data are shown as mean ± SD. Black dotted lines indicate the limit of detection (LOD) or lower limit of confidence (LLOC). All comparisons were statistically assessed using Brown-Forsythe and One-Way ANOVA and Dunnett T3 correction for multiple comparisons (two-tailed) (all not significant).

The elderly population is of special concern when it comes to COVID-19, due to their increased vulnerability and diminished vaccine efficacy. Hence, we assessed the potential of our MCMV^S^-based vaccine in aged mice. Specifically, we immunized aged K18-hACE2 mice at 14–16 months of age either with MCMV^S^ or with MCMV^WT^ as a control vector (**Figure 3A**). The neutralization capacity of sera was examined at 6 and 12 weeks, as well as up to 24 weeks post-immunization. Neutralizing responses generally increased over time (**Figure 3B**). This especially affected the SARS-CoV-2 Omicron BA.1 or BA.4/5 specific antibodies, where a neutralization response was detected in a few mice only at 6 weeks after immunization. However, cross-reactive titers against these variants significantly increased over time against both Omicron subvariants, with approximately half of the animals exhibiting robust titers at 6 months post-vaccination (**Figure 3B**).

Repeated antigen exposure enhances somatic hyper-mutation and affinity maturation processes in B cells, yielding high-affinity antibodies that efficiently bind to antigens (41–43) and improves virus neutralization efficacy. Since MCMV-based vaccines stimulated robust antibody responses with increasing neutralization capacity (17), we considered that these functional responses may be driven by improved affinity maturation, or by polyclonal B-cell expansion and a total increase in antibody titers. Hence, we longitudinally quantified SARS-CoV-2 spike-specific total IgG levels upon vaccination. Kinetic measurements revealed no significant differences in total IgG levels, against both the SARS-CoV-2 Index and the Omicron BA.1 variant (**Figure 3C**). Serum samples from control mice that underwent mock immunization were typically below the detection threshold (data not shown). We next tested IgG binding avidity by exposing sera to various concentrations of sodium thiocyanate (NaSCN), as described previously (17). We observed an increase in avidity over time, from 6 and 12 to 24 weeks post-immunization (**Figure 3D**). Consistent levels of total anti-S IgG alongside a concurrent rise in binding affinity within the same sera may indicate that the increase in neutralization was driven primarily by affinity maturation.

Following on from this, independent cohorts of aged mice were tested up to 16 months following low-dose immunization (2 × 10^5^ PFU) of MCMV^S^ to assess durability of vaccine-mediated immunogenicity in the context of reduced vaccine dosage and an extended time-frame (**Supplementary Figure 5A**). Even when immunized with a lower dose, MCMV^S^-immunized mice displayed high frequencies of SARS-CoV-2 spike peptide (VNFNFNGL)-specific CD8^+^ T cells over a period of 270 days (**Supplementary Figure 5B**). Monitoring effector-memory cells (T_EM_; CD127^+^/KLRG1^-^) and effector-like cells (CD127^-^/KLRG1^+^) revealed a constant decrease in effector-like cells and a stable increase in T_EM_ cells (**Supplementary Figures 5C, D**). Likewise, anti-Spike IgG responses to SARS-CoV-2 increased following MCMV^S^ immunization (**Supplementary Figure 5E**).

Analysis of specific T-cell responses revealed a higher frequency of SARS-CoV-2 spike (VNFNFNGL)-specific CD8^+^ T cells, or after stimulation with a peptide pool covering the Index and Omicron variants of the spike protein (**Supplementary Figures 5F–H**). Comparing specific T-cell responses against SARS-CoV-2, it is notable that reactivation after stimulation with the Omicron peptide pool was the highest (**Supplementary Figure 5I**). Spike-specific CD8^+^ T-cell responses in the spleen remained detectable, with a mean frequency of approximately 12% of VNFNFNGL-specific cells in the CD8^+^ compartment after 270 days and 9% after 16 months post-immunization (**Supplementary Figures 5J, L**), and exhibiting a predominant effector phenotype (**Supplementary Figures 5K, M**).

### Aged MCMV^S^-immunized mice show long-lasting protection against heterologous SARS-CoV-2 challenge

**Figure 4:**
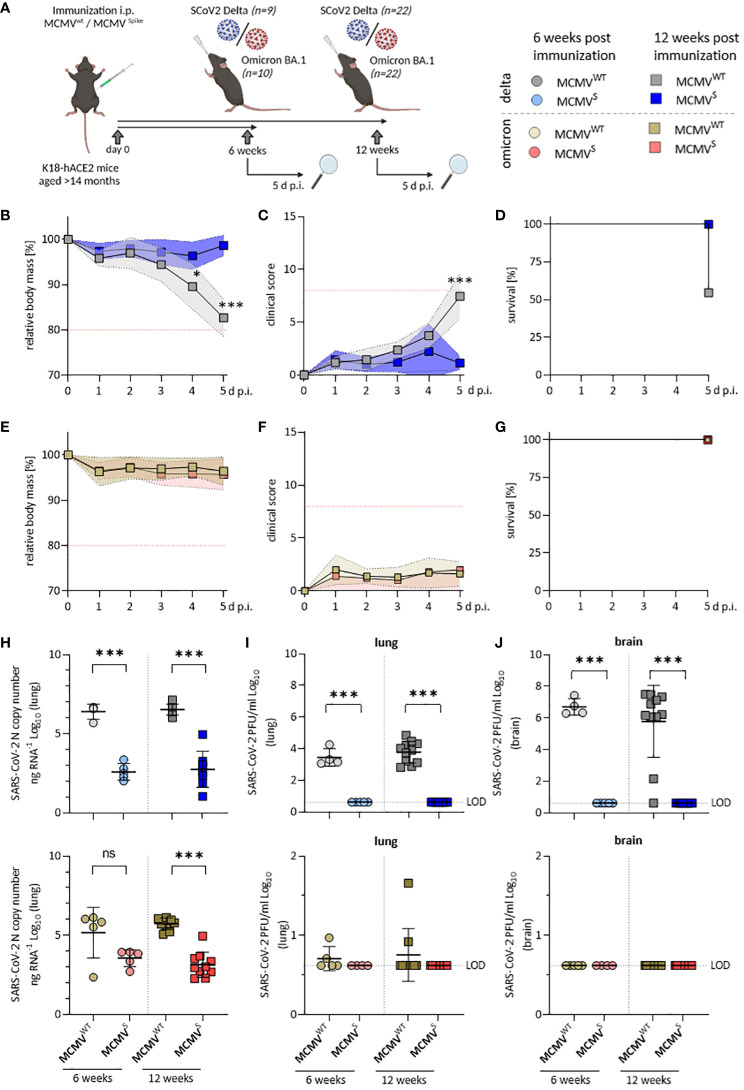
Aged MCMV^S^-immunized mice show long-lasting protection against SARS-CoV-2 infection. **(A)** Schematic representation of the experimental setup. Created with BioRender.com. **(B)** Relative body mass, **(C)** clinical scoring, and **(D)** survival of mice challenged with SARS-CoV-2 Delta at twelve weeks post-immunization (MCMV^WT^ n=11, MCMV^S^ n=9). **(E)** Relative body mass, **(F)** clinical scoring, and **(G)** survival of mice challenged with SARS-CoV-2 Omicron BA.1 at twelve weeks post-immunization (MCMV^WT^ n=10, MCMV^S^ n=10). **(H)** Viral loads as SARS‑CoV-2 N gene copy number as copy number/ng RNA Log10 in murine lungs five days after challenge with SARS-CoV‑2 Delta (top) or Omicron BA.1 (bottom) at six weeks (MCMV^WT^ n=4-5, MCMV^S^ n=5), and twelve weeks (MCMV^WT^ n=10-11, MCMV^S^ n=8-10) post-immunization. **(I)** Infectious virus titers in murine lungs and brains **(J)** five days after challenge with SARS-CoV‑2 Delta (top) or Omicron BA.1 (bottom) at six weeks (MCMV^WT^ n=4-5, MCMV^S^ n=5), and twelve weeks (MCMV^WT^ n=10-11, MCMV^S^ n=8-10) post-immunization. Data in **(H–J)** are shown as mean ± SD. Red dotted lines in **(B, C, E, F)** indicate the maximal acceptable burden for animal experiments. The humane endpoint (>20% reduction in body mass **(B, E)** or a clinical score of ≥8 **(C, F)**) was pre-defined by authorized animal trial permits. Black dotted lines indicate the limit of detection (LOD). For survival analyses **(D, G)**, a log-rank (Mantel-cox) test was used to assess statistical significance. Statistical significance for longitudinal assessments **(B, C, E, F)** was calculated with Greenhouse-Geisser corrected Two-Way ANOVA and Tukey post-hoc testing. All other comparisons **(H–J)** were generally statistically assessed using Brown-Forsythe and One-Way ANOVA and Dunnett T3 correction for multiple comparisons (two-tailed). Statistical assessments in panels **(I, J)**, where some groups were uniformly negative, were assessed by ordinary One-Way ANOVA with Sídák correction for multiple comparisons. (* p < 0.05, *** p < 0.001, ns = not significant).

We subsequently assessed protection of a single-dose MCMV^S^ vaccination against SARS-CoV-2 challenge in aged mice. Aged K18 hACE2 mice (>14 months) were challenged with 2 × 10^3^ PFU of SARS-CoV-2 Delta to identify protection against a highly virulent variant, or SARS-CoV-2 Omicron BA.1 to test a variant with strong immune escape, at 6 or 12 weeks post-immunization (**Figure 4A**). Mice immunized with MCMV^S^ were fully protected against body mass loss (**Supplementary Figure 6A**), clinical signs (**Supplementary Figure 6B**), and death (**Supplementary Figure 6C**) following SARS-CoV-2 Delta challenge at 6 weeks after MCMV^S^ immunization when compared to MCMV^WT^-controls. Moreover, protection against body mass reduction (**Figure 4B**), clinical manifestations of disease (**Figure 4C**), and death (**Figure 4D**) was maintained for up to 12 weeks post-immunization when challenged with SARS-CoV-2 Delta variant, while mock-vaccinated mice exhibited severe illness, and 45.5% (5/11) reached the humane endpoint by day 5 post-infection (**Figure 4D**). Mice challenged with SARS-CoV-2 Omicron BA.1 at 20 weeks post-immunization demonstrated no body mass loss (**Figure 4E**) and exhibited only mild clinical symptoms (**Figure 4F**), irrespective of immunization status, consistent with our previous findings (**Figure 2**).

Vaccine efficacy was further evaluated by quantifying the SARS-CoV-2 RNA load (**Figure 4H**) and infectious titers in the lungs (**Figure 4I**) and brain (**Figure 4J**) at 5 d p.i. Analyses of viral load in SARS-CoV-2 Delta-challenged mice revealed a significant, >1,000-fold reduction in lungs (**Figure 4H**, top) and brains (**Supplementary Figure 6D**) at both 6 and 12 weeks post-immunization. We also observed a significant reduction of SARS-CoV-2 viral RNA in lungs (**Figure 4H**, bottom) of mice challenged with the Omicron BA.1 variant at 12 weeks post-immunization and a clear tendency of reduced viral loads at 6 weeks post-immunization. Only low levels of SARS-CoV-2 Omicron BA.1 mRNA could be detected in brains of infected mice, even in mock-vaccinated mice (**Supplementary Figure 6E**).

MCMV^S^-immunized mice harbored no detectable infectious virus particles in lungs (**Figure 4I**) and brains (**Figure 4J**) at 5 days post-challenge with SARS-CoV-2 Delta, indicating efficient and fast clearance in immunized animals. Only few mock-immunized SARS-CoV-2 Omicron BA.1-infected animals showed infectious titers, in line with our previous observations (44) and all MCMV^S^-immunized animals were SARS-CoV-2 Omicron BA.1 negative at the time of sampling. Taken together, MCMV^S^ protected aged mice from disease after SARS-CoV-2 Delta challenge up to 3 months post-vaccination and reduced viral loads following challenge with both SARS-CoV-2 Delta and the antigenically distinct Omicron BA.1 variant.

### MCMV^S^ protects against subclinical inflammation following SARS-CoV-2 Omicron BA.1 infection

**Figure 5:**
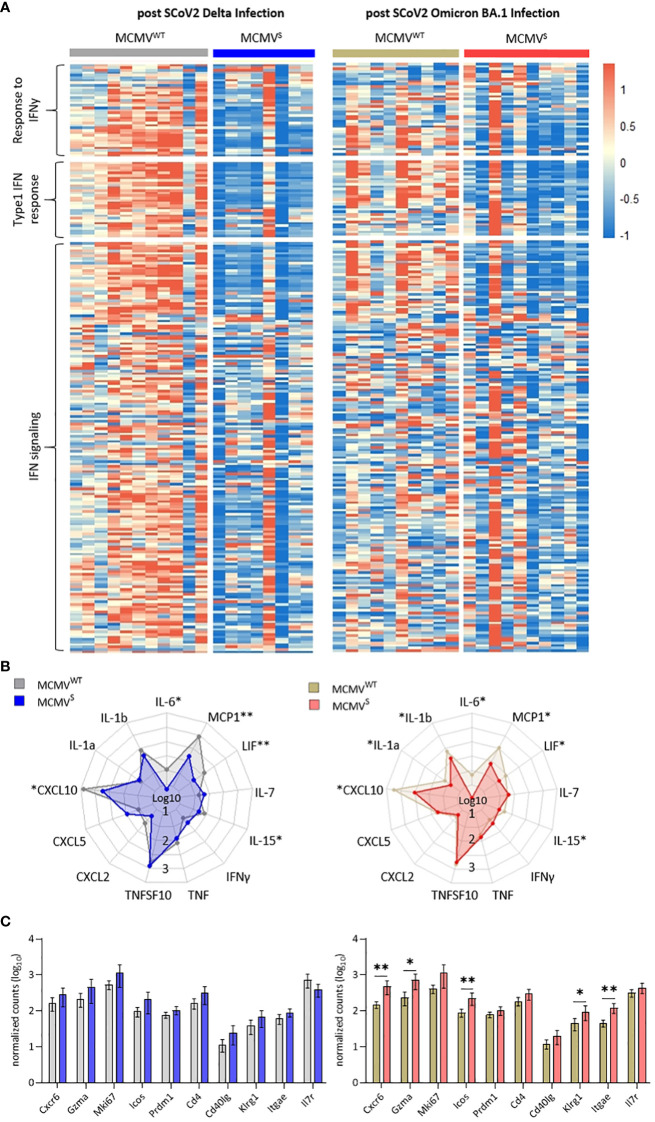
MCMV^S^ protects against subclinical inflammation following SARS-CoV-2 Omicron BA.1 infection. **(A)** Heat maps of differently expressed genes in response to infection involved in the response to IFNy, type 1 IFN response, and related to IFN signaling, based on (45). Differentially expressed genes of mice infected with SARS-CoV-2 Delta (left) or Omicron BA.1 (right) that were treated with MCMV^WT^ (Delta n=11, Omicron BA.1 n=10) or immunized with MCMV^S^ (Delta n=8, Omicron BA.1 n=10) are shown as analyzed by bulk RNA sequencing of the lung tissue at day five post-infection. Columns represent different animal samples and rows represent different genes. Shown are z-scores of DESeq2-normalized data and color scale ranges from red (10 % upper quantile) to blue (10 % lower quantile), showing up- or downregulation in expression of the selected genes, respectively. **(B)** Spider plot of differentially expressed cytokines between the MCMV^WT^- or MCMV^S^-immunized animals. Values are DESeq2-normalized data on a log10 scale represented for each gene analyzed. **(C)** Bar graph of differentially expressed genes involved in T-cell activation and proliferation based on (46). Shown are DESeq2-normalized values on a log10 scale. The group sizes are identical to panel **(A)**. Data in **(C)** are shown as mean ± SD. Statistical assessments were performed using multiple Welch’s t-test corrected for multiple comparisons following Holm-Sidák. (* p < 0.05, ** p <0.01).

We used bulk RNA sequencing of lung homogenates to investigate gene expression patterns associated with inflammation in lung samples after challenge with SARS-CoV-2 Delta or Omicron BA.1 at 12 weeks after immunization (as shown in **Figure 4**). We observed a consistent difference in expression levels of genes involved in pathogen response and inflammation between the vaccinated mice and their mock-vaccinated controls. The expression of genes related to interferon-gamma (IFNγ) and the type I IFN response, as well as the IFN signaling cascade, were clearly reduced in vaccinated mice upon both SARS-CoV-2 Delta and Omicron BA.1 challenge (**Figure 5A**). These data were in line with the reduction in symptoms observed upon SARS-CoV-2 Delta challenge in mice and indicated that SARS-CoV-2 Omicron BA.1 infection induces a subclinical inflammation in mice, which was clearly reduced following vaccination. Cytokines that play crucial roles in inflammatory processes and are suspected to contribute to immunopathology were analyzed in more depth. Several cytokines (IL-6, MCP-1, LIF, IL-15, and CXCL10) were significantly elevated in the mock- compared to the MCMV^S^-vaccinated animals after SARS-CoV-2 Delta (**Figure 5B**, left) and Omicron BA.1 (**Figure 5B**, right) challenge. In addition, IL-1a and IL-1b were significantly elevated in the mock- compared to the MCMV^S^-vaccinated animals only after SARS-CoV-2 Omicron BA.1 challenge (**Figure 5B**, right). As expected by clinical presentation, cytokine expression in mock-immunized animals was higher among the animals challenged with the SARS-CoV-2 Delta variant than in the SARS-CoV-2 Omicron BA.1-challenged ones. Cytokine expression was similarly reduced among MCMV^S^-immunized mice. In contrast to inflammatory markers, T-cell markers associated with the activation (Klrg1, Icos, Cd40lg, Gzma, and Cd4), proliferation (Mki67), and memory formation (Il7r) of T-cell responses post-vaccination or markers related to tissue retention or homing (Cxcr6, Itgae, and Prdm1), as previously shown (46), were upregulated, or showed a tendency to be upregulated in MCMV^S^-immunized mice compared to their controls upon SARS-CoV-2 Delta and Omicron BA.1 infection (**Figure 5C**).

Cell-type deconvolution of bulk RNA sequencing data from lung homogenates and blood indicated significant changes in cell proportions following SARS-CoV-2 challenges in MCMV^S^-immunized mice. After the Delta challenge, the percentage of B cells was higher, while the percentages of monocytic macrophages and neutrophils were lower compared to MCMV^WT^ mock-immunized controls (**Supplementary Figure 7A**). Similarly, following the Omicron BA.1 challenge, the proportion of neutrophils was significantly reduced, whereas both T cells and NK cells significantly increased in MCMV^S^-immunized mice compared to those immunized with MCMV^WT^ (**Supplementary Figure 7B**).

In summary, our findings indicate that vaccination with MCMV^S^ not only leads to a strong immune response but also protects mice against subclinical inflammation caused by the SARS-CoV-2 Omicron BA.1 variant.

## Discussion

Several vaccines have been developed to combat the SARS-CoV-2 pandemic. Nevertheless, immune waning remains an unresolved challenge (6–8, 47), and vaccines resulting in lasting and broad protection against SARS-CoV-2 variants remain unavailable. Viral vectors that were authorized early in the COVID-19 pandemic, such as Vaxzevria (ChAdOx1-S) and the COVID-19 vaccine from Janssen (Ad26.COV2.S) (48, 49), provide immune protection that is relatively short-lived, which is also true for the mRNA-based vaccines (50). CMV has received attention as a potential vaccine vector tool because it induces strong and lasting T-cell immunity (26). While HCMV seroprevalence is estimated to exceed 90% in some geographic areas, the pre-existing immunity to CMV does not hinder vaccine responses and protection in studies with rhesus monkeys (27, 51), arguing that CMV vectors may be used in CMV seropositive populations. MCMV shares structural and functional homologies with HCMV and allows *in vivo* analyses in the natural host. We show here, using the murine CMV vaccine vector model, that CMV-based vectors provide durable and protective immunity upon a single-dose immunization in mice. Hence, our data may indicate that a CMV-based vaccine may provide durable and broad immune protection against COVID-19 in humans.

Neutralizing antibody responses correlate with vaccine effectiveness (52) and our vaccine MCMV^S^ elicits neutralizing antibody responses in mice whose affinity and neutralizing capacity increase over time when assessed for 6 months following immunization (17). Here, we show that, in mice, MCMV^S^ protects against disease upon infection with the SARS-CoV-2 Beta variant and against subclinical inflammation upon infection with the SARS-CoV-2 Omicron BA.1 variant, with neutralization titers increasing for at least 6 months post-vaccination. This feature is unique for this vector and superior to other vaccine formulations, which require repeated booster administrations to avoid immune waning over time (48, 49, 53, 54). Considering that neutralizing antibodies against SARS-CoV-2 wane at similar rates upon infection or vaccination with currently used vaccines (9, 47, 55–57), our vaccine might provide an immunity that is even better than that induced by infection. Similarly, T-cell responses against the SARS-CoV-2 spike-encoded epitope VNFNFNGL improved over time and maintained an effector-memory phenotype. A single CD8^+^ T-cell epitope encoded by an MCMV vector was sufficient to elicit protective immunity against Ebola or herpes virus (28, 58). While cellular immunity may contribute to protection, this needs to be dissected in targeted approaches, by inducing T-cell responses in absence of humoral immunity and in functional assays upon *in vitro* antigenic re-stimulation.

Interestingly, even the relatively weak neutralizing antibody responses against the SARS-CoV-2 Omicron BA.1 variant were associated with protection against subclinical inflammation and lowered viral loads in the lungs. One may speculate that T-cell immunity elicited by MCMV^S^-vaccination protected these animals. Similar phenomena were documented in preclinical studies with rhesus macaques or in clinical studies, where study subjects were protected although no neutralizing antibodies were observed (59–61). However, we cannot exclude the possibility that memory B lymphocytes in MCMV^S^-immunized mice responded to the challenge by rapidly generating neutralizing antibodies upon challenge, thus limiting virus replication and protecting the host. Indeed, we observe that neutralizing antibody titers rapidly increase following challenge at 20 weeks following immunization (**Figure 2I**, **Supplementary Figures 4C, D**). Finally, it may also be possible that the concerted action of these two lymphocyte lineages protected the vaccinated mice against challenge. To differentiate between these scenarios, one may use MCMV vectors eliciting only T-cell responses against SARS-CoV-2 spike epitopes, or mice lacking T cells. However, such experiments would go beyond the scope of this study.

In this study, we showed that even a SARS-CoV-2 spike antigen that cannot bind to the target ACE2 receptor in vaccinated mice may still provide robust immune protection against disease. Namely, the spike antigen from the Index variant of SARS-CoV-2, which was cloned into our vaccine vector, cannot bind to the murine ACE2 receptors present in BALB/c mice that we have used in **Figure 2** (62). Nevertheless, MCMV^S^ protected BALB/c mice against disease following SARS-CoV-2 Beta challenge and reduced viral loads following SARS-CoV-2 Omicron BA.1 challenge, which both encode an N501Y mutation and thus engage the mACE2 and may replicate in mice. Some studies reported that the binding of the S1 subunit of the SARS-CoV-2 spike protein to ACE2 on endothelial cells may affect endothelial barrier integrity and cardiac activity (63–65), which would be excluded in such approaches. While we observed no adverse effects in hACE2 transgenic mice immunized in the long term with our vector, arguing against such adverse effects (or other side effects, unrelated to ACE2 biding), our data demonstrate that SARS-CoV-2 spike formulations may protect against SARS-CoV-2 infection in the absence of ACE2 receptor engagement by the vaccine-encoded SARS-CoV-2 spike, which was a concern in light of low levels of SARS-CoV-2 spike antigen persistence in the host.

SARS-CoV-2 Omicron BA.1 infection of aged K18 hACE2 mice did not result in clinical manifestation of disease. Therefore, we applied bulk-RNA sequencing on lungs from these mice that allowed us to observe subclinical patterns of inflammation following SARS-CoV-2 Omicron BA.1 infection. Importantly, no signs of subclinical inflammation could be observed in MCMV^S^-immunized mice, enabling us to characterize and quantify the level of protection by our vaccine against subclinical inflammation in the absence of clinical disease (**Figures 4**, **5**). We propose that bulk-RNA sequencing could be applied in future studies to assess vaccine-mediated protection in Omicron BA.1-challenged mice.

The data presented here demonstrate that an MCMV-based vaccine candidate expressing the full-length SARS-CoV-2 Index spike protein is highly immunogenic and protective in mice. We demonstrated that a single dose of the MCMV^S^ may protect not only young, but also aged mice against a potentially lethal dose of SARS-CoV-2 Beta and Delta, and that protective effects such as reduced subclinical inflammation or reduced viral lung titers could also be observed following SARS-CoV-2 Omicron BA.1 challenge. Our approach provided evidence for long-lasting immunity, especially in terms of T-cell responses. Future research needs to focus on a head-to-head comparison of MCMV-based vaccines with other COVID-19 vaccine formulations, on the length of protection and administration routes, paving the way towards clinical trials with CMV vectors. Once this vaccine technology is applied in non-murine hosts, a thorough assessment of safety and potential side effects will be required. We propose a radical departure from the conventional vaccines against COVID-19 that provide only short-term protection and require continuous boosters and frequent adaptations of the antigen in the vaccine to circulating spike variants. This study demonstrates that, in principle, such approach is feasible.

## Material and methods

### Cell culture and viruses

Vero E6 (CRL-1586) and M2-10B4 cells (ATCC CRL-1972) were cultured as described previously (17). Caco-2 cells (ACC 169) were purchased from DSMZ (Braunschweig, Germany) and were cultured in DMEM (Gibco, NY, USA) supplemented with 20% fetal bovine serum (FBS), 2 mM L-glutamine, 100 IU/mL penicillin, and 100 μg/mL streptomycin. The FI strain of SARS-CoV-2 (GISAID database ID: EPI_ISL_463008) was described previously as a D614G variant (66) and was passaged on Caco-2 cells in the biosafety level 3 (BSL3) laboratory at HZI. A clinical isolate of the SARS-CoV-2 B.1.617.2 (Delta) variant was isolated at the Fran Mihaljevic clinical center in Zagreb and propagated as previously described (66).

SARS-CoV-2 genome sequences are available on GISAID and GenBank under the following accession numbers: SARS-CoV-2 B.1.351 (Beta) FFM-ZAF1/2021 (GenBank ID: MW822592) (67) and SARS-CoV-2 B.1.1.529 (BA.1) FFM-ZAF0396/2021 (EPI_ISL_6959868; GenBank ID: OL800703) (68). MCMV^WT^ refers to the BAC-derived molecular clone of MCMV (pSM3fr-MCK-2fl clone 3.3) (69). MCMV^S^ was generated by *en passant* mutagenesis, as described previously (17). Briefly, the codon-optimized spike protein of the Index variant replaced the coding sequence of the viral ie2 protein.

The expression vector for the SARS-CoV-2 spike protein of Omicron (BA.1) (based on isolate hCoV-19/Botswana/R40B58_BHP_3321001245/2021; GISAID Accession ID: EPI_ISL_6640919) and BA.4/5 (based on isolate hCoV-19/England/LSPA-3C01A75/2022) was generated by Gibson assembly as described previously (70) and then subsequently introduced in pseudo-type VSV backbone that lacks VSV glycoprotein G (VSV-G) (71). A plasmid encoding the spike protein of SARS-CoV-2 Beta (B.1.351) has been previously reported (17).

### Virus stock generation and plaque assay

BAC-derived MCMV was reconstituted by transfection of BAC DNA into NIH-3T3 cells (ATCC CRL-1658) using FuGENE HD transfection reagent (Promega, WI, USA) according to the manufacturer’s instructions. Transfected cells were cultured until viral plaques appeared and passaged five times in M2-10B4 cells before virus stock production. First, supernatants of infected M2-10B4 cells were collected and infected cells were pelleted (5,000 × *g* for 15 min). The resulting cell pellets were homogenized in DMEM supplemented with 5% FBS and cell debris was removed by centrifugation (12,000 × *g* for 10 min). Collected supernatants were resuspended in VSB buffer (0.05 M Tris-HCl, 0.012 M KCl, and 0.005 M EDTA, adjusted to pH 7.8) and then concentrated by centrifugation through a 15% sucrose cushion in VSB buffer (23,000 × *g* for 1.5 h). The resulting pellet was resuspended in 1–1.5 mL of VSB buffer and briefly spun down, and supernatants were aliquoted and kept at −80˚C. BAC-derived mutant MCMV^S^ were propagated on M2-10B4 cells and concentrated by sucrose density gradient centrifugation.

SARS-CoV-2 D614G was generated and viruses were quantified by plaque assays as described before (17) with the modification that Caco-2 cells were used for virus production. SARS-CoV-2 Beta, Delta, and Omicron BA.1 stocks were generated as described previously and titers were determined by plaque assay (68).

Pseudo-typed viruses were harvested as described before (17, 70). In brief, 293T cells were transfected with expression plasmids (pCG1) encoding different spike proteins of SARS-CoV-2 variants by using the calcium-phosphate method. At 24 h post-transfection, the medium was removed and cells were inoculated with a replication-deficient VSV vector lacking its glycoprotein and coding instead for an enhanced green fluorescent protein (GFP) (kindly provided by Gert Zimmer, Institute of Virology and Immunology, Mittelhäusern, Switzerland). Following 1-h incubation at 37°C, the cells were washed with PBS, and culture media containing anti-VSV-G antibody (culture supernatant from I1-hybridoma cells; ATCC CRL-2700) were added. The pseudo-type virus was harvested at 16–18 h post-infection.

### Virus *in vivo* infection

K18-hACE2 mice were obtained from Jackson Laboratories and bred in the core animal facility of the Helmholtz Center for Infection Research, Braunschweig. BALB/c mice were purchased from Envigo (IN, USA). All animals were housed under specific pathogen-free (SPF) conditions at HZI during breeding and infection. All animal experiments were approved by the Lower Saxony State Office of Consumer Protection and Food Safety, license number: 33.19-42502-04-20/3580.

K18-hACE2 mice (2 to 6 months old and >14 months) were intraperitoneally (i.p.) immunized with 10^6^ PFU of recombinant MCMV^S^ or MCMV^WT^ diluted in PBS or treated with PBS (200 μL i.p. per animal). BALB/c mice (4–7 months old) were i.p. immunized with 2 × 10^5^ PFU of recombinant MCMV^S^ or MCMV^WT^. All mice were weekly or bi-weekly monitored and scored for their health status after vaccination. Blood was isolated at indicated time points by puncture of the retrobulbar plexus or by cardiac bleeding upon death.

SARS-CoV-2 challenge experiments were performed in the HZI BSL3 laboratory essentially as described (72) with the following modifications: K18-hACE2 mice were intranasally (i.n.) infected with 2 × 10^3^ PFU of SARS-CoV-2 D614G, Delta, or Omicron BA.1, while BALB/c mice were challenged with 6 × 10^4^ PFU of SARS-CoV-2 Beta or Omicron BA.1. SARS-CoV-2-infected mice were monitored for body mass loss and clinical status at least daily, according to the animal permit.

### Clinical scoring system

SARS-CoV-2-challenged animals were scored daily to monitor any signs of disease development. Animals were scored based on five criteria: spontaneous/social behavior, fur, fleeing behavior, posture, and body mass loss. Each score indicates the following: no signs of symptoms (score = 0), mild and/or sporadic symptoms (score = 1), moderate and/or frequent symptoms (score = 2), and severe symptoms with a clear sign of heavy suffering (score = 3). Reduction in body mass was scored as follows: ≤1% (score = 0), 1%–10% (score = 1), 10%–20% (score = 2), and >20% (score = 3). Mice with a score of 3 in one criterion, or an overall score of ≥8, were removed from the experiments.

### Organ harvest

Animals were euthanized by CO_2_ inhalation; blood was collected from the heart, kept at room temperature (RT) for 30–60 min until clotting occurred, and then stored at 4°C. For further analysis, serum was isolated by centrifugation. The trachea, lungs, heart, spleen, stomach, and brain were harvested at 3, 5, or 7 days post-SARS-CoV-2 challenge. In animals that reached humane endpoints before 7 days post-challenge, organs were harvested on the day of euthanasia. Solid organs were weighed and homogenized in bead-containing lysis tubes (Lysis tube E, Analytik Jena) in 500 µL or 1,000 μL of PBS with an MP Biomedical FastPrep 24 Tissue Homogenizer (MP Biomedicals, CA, USA) (full speed, 2 × 20 s) and stored at −80°C. Lung homogenates designated for RNA isolation or bulk sequencing were mixed with Trizol reagent (Invitrogen) at a 1:3 ratio and snap-frozen in liquid nitrogen. All centrifugation steps were carried out at 10,000 × *g* for 10 min at 4°C.

### RNA isolation and viral load analyses

RNA isolation was performed with the RNeasy RNA isolation kit (Quiagen), or innuPrep Virus TS RNA kit (Analytik Jena), according to the manufacturer’s protocols. Shortly, for the first experiments of infected K18-hACE2 mice, challenged with D614G, the RNeasy RNA isolation kit was used, by adding 250 μL of organ homogenates in 750 μL of Trizol, and subsequently centrifuged at 16,000 × *g* for 3 min. The resulting supernatants were carefully collected and washed with the same volume of 70% ethanol. The mixed solution was transferred into a collection tube and centrifuged at 10,000 × *g* for 30 s. After decanting the flow-through, the column was washed once with 700 μL of RW1 wash buffer and twice with 500 μL of RPE buffer. Lastly, 40 μL of nuclease-free water was added to the column for RNA elution. For the remaining experiments including the BALB/c and K18-hACE2 mice challenged with the SARS-CoV-2 variants RNA extraction was conducted using the “innuPrep Virus TS RNA kit” (Analytik Jena) following the manufacturer’s instructions. In brief, Trizol-inactivated samples were combined with an equal volume of lysis solution CBV, which contained carrier RNA and Proteinase K. This mixture was then incubated at 70°C for 10 min. After the lysis step, samples were blended with two sample volumes of isopropanol. Subsequently, the samples were applied to the provided spin filters, washed with washing buffer (LS), and rinsed with 80% (v/v) ethanol. RNA was finally eluted in 60 µL of RNase-free water and stored at −80°C after determining the RNA concentration using a NanoDrop (Thermo Scientific NanoDropOne).

Eluted RNAs were analyzed further to assess viral RNAs in the given organs by quantitative reverse transcription polymerase chain reaction (RT-qPCR). The reaction was performed with a total volume of 20 μL containing 2 μL of sample RNAs or positive control RNAs, 5 μL of TaqPath 1-step RT-qPCR Master Mix with ROX reference dye, and 1.5 μL of probe/primer sets. The 2019-nCoV RUO kit was used to detect SARS-CoV-2 RNAs [Integrated DNA Technologies (IDT), USA], and Taqman Rodent GAPDH control reagents (Thermo Fisher Scientific, USA) were used for endogenous GAPDH RNAs. For absolute viral RNA quantification, a standard curve was generated by serially diluting a SARS-CoV2 plasmid with the known copy numbers 200,000 copies/μL (2019-nCoV_N_Positive Control, #10006625, IDT, USA) at a 1:2 ratio in all PCR analyses, with a quantitation limit of 20 copies of the plasmid standard in a single qPCR reaction. The viral RNA of each sample was quantified in triplicate, and the mean viral RNA was calculated by the standard. RT-qPCR was performed using the StepOnePlusTM Real-Time PCR system (Thermo Fisher Scientific, USA) according to the manufacturer’s instructions. SARS-CoV-2 N-gene copy numbers were normalized to total RNA input and to rodent GAPDH copy numbers.

### Detection of infectious SARS-CoV-2 titers by plaque assay

Lung and brain organ homogenates were serially diluted in DMEM (Gibco, NY, USA) supplemented with 5% FBS, 100 IU/mL penicillin, and 100 μg/mL streptomycin. Sample dilutions (100 μL) were transferred onto confluent VeroE6 cells in a 96-well format. After inoculation for 1 h at 37°C, the inoculum was removed and 1.5% methylcellulose in MEM supplemented with 5% FBS, 2 mM L-glutamine, 100 IU/mL penicillin, and 100 μg/mL streptomycin was added to the cells. The infected cells were incubated at 37°C for 48 h before inactivation with a 4% formalin solution in PBS for 10 min at RT. The fixed cells were subjected to immunofluorescent staining against the SARS-CoV-2 nucleocapsid (N) protein. Briefly, fixed cells were permeabilized with 0.1% Triton X-100 (Sigma-Aldrich, MA, USA) for 10 min at RT and blocked with 1% BSA (Sigma-Aldrich, MA, USA) in PBS for 30 min at RT. Thereupon, cells were incubated with a monoclonal anti-SARS-CoV-2 N protein antibody (Abcalis, AB84-E02, 10 μg/mL) for 30 min at RT. After washing three times with PBS with 0.05% Tween20 (PBS-T), a secondary antibody anti-mouse IgG conjugated with Alexa488 (Cell Signaling Technology, #4408, 1:500) was added for 30 min at RT. After washing three times with PBS-T, the stained cells were visualized using Incucyte S3 (Sartorius; GUI software versions 2019B Rev1 and 2021B). Stock virus that was used for *in vivo* challenges was used as positive control for this assay.

### Flow cytometric quantification of VNFNFNGL-specific CD8^+^ T cells

Peripheral blood was harvested and red blood cells were removed by short osmotic shock. Thereupon, lymphocytes were stained with SARS-CoV-2 spike-derived VNFNFNGL-specific tetramers (kindly provided by Ramon Arens, Leiden University) for 30 min at RT. Subsequently, cells were stained with fluorescent-labeled antibodies against CD3 (17A2, eBiosciences, CA, USA), CD4 (GK1.5, BioLegend, CA, USA), CD8a (53-6.7, BD Bioscience, 528 CA, USA), CD44 (IM7, BioLegend, CA, USA), CD11a (M17/4, BioLegend, CA, USA), CD62L (MEL-14, BioLegend, CA, USA), CD127 (SB/199, BioLegend, CA, USA), and KLRG1 (2F1, BioLegend, CA, USA) for 30 min at 4°C. Dead cells that were identified by 7-AAD viability staining solution (BioLegend, CA, USA) were excluded from all analyses. The labeled cells were analyzed by flow cytometry (BD LSRFortessaTM Cell Analyzer) and subsequent analyses were done in detail in FlowJo Software v10. For the experiments where mice were immunized with a low dose (2 × 10^5^ PFU), samples were isolated and stained as follows: Splenocytes were isolated using a standard protocol. Briefly, mice were sacrificed, and spleens were harvested and homogenized; this was followed by erythrocyte lysis. Blood samples were collected from a saphenous vein, followed by erythrocyte lysis. After leukocyte isolation, Fc receptors were blocked with 2.4G2 mAb to reduce nonspecific staining. For surface staining, the following antibodies were used: anti-CD45.2 eFluor 506 (clone: 104, 1:200), anti-CD3 BV786 (clone: 145-2C11; 1:100, BD), anti-CD8α SB600 (clone: 53–6.7; 1:400), anti-CD44 A700 (clone: IM7, 1:100), anti-CD11a PerCP-eF710 (clone: M17/4, 1:100), anti-KLRG1 PE (2F1; 1:200),anti-CD127 PerCP-eF710 (SB/199; 1:100), and anti-CD62L PE-Cy7 (MEL-14; 1.400). Spike-specific tetramer (539VNFNFNGL546; H-2Kb) was synthesized by the National Institutes of Health tetramer core facility. Fixable Viability Dye eFluor-780 (1:1,000) was used to exclude dead cells. IFN-γ, TNF-α, and IL-2 production by CD8 T cells was stimulated by incubation with antigenic peptides as previously described (73) and examined by intracellular staining. For intracellular staining, an intracellular fixation and permeabilization buffer set (eBioscience) was used along with the following antibodies: anti-IFN-γ FITC (XMG1.2, 1:100), anti-TNF-α PE-eF610 (MP6-XT22, 1:100), and anti-IL-2 APC (JES5-5H4; 1.100). All antibodies were purchased from eBioscience, unless stated otherwise. *In vitro* stimulation assay has been performed using an overlapping pool of 158 peptides PepMix SARS-CoV-2 Spike (JPT Peptide Technologies) and H2-Dd restricted S protein epitope KNKCVNFNF (GenScript). Data were acquired using FACSAriaIIu (BD Biosciences) and analyzed using FlowJo v10 (TreeStar) software.

### Detection of anti-spike antibodies in mouse sera

ELISA (enzyme-linked immunosorbent assay) was used to detect SARS-CoV-2 spike-specific IgG responses in murine sera. As control, sera from mock-immunized mice were included. Proteins spanning the S1–S2 domain of either the SARS-CoV-2 Index (Wuhan) or B.1.1.529 variant (Omicron) were used as antigens, which were produced in insect cells as described before (6, 72), in a baculovirus-free system. For antigen coating, 100 ng/well of SARS-CoV-2 S1–S2 protein in carbonate buffer (50 mM NAHCO_3_/Na_2_CO_3_, pH 9.6) was immobilized overnight at 4°C in 96-well plates (Greiner Bio-One). ELISA plates were blocked with 2% MPBS-T [2% (w/v) skim milk powder and 0.05% Tween20 in PBS] for 1 h at RT and washed with H_2_O containing 0.05% Tween20 using EL405 S washer (BioTek). Murine sera were diluted 1:100 and titrated to a final dilution of 1:6,400 and incubated for 1 h at RT. After washing as described above, the serum IgG response was detected using horseradish peroxidase (HRP)-conjugated goat-anti mouse IgG antibody (A0186, Sigma) (1:42,000 diluted in 2% MPBS-T) and plates were washed again after incubation at RT for 1 h. Binding was visualized using tetramethylbenzidine (TMB) substrate {19 parts TMB A solution [30 mM potassium citrate; 1% (w/v) citric acid (pH 4.1)] and 1 part TMB B solution [10 mM TMB; 10% (v/v) acetone; 90% (v/v) ethanol; 80 mM H_2_O_2_ (30%)]} and developed for 15 min at RT. The reaction was stopped using 0.5 M/1 N sulfuric acid and the absorbance was measured at 450 nm with subtracting reference at 620 nm using the ELISA plate reader (Epoc, BioTek). EC_50_ was analyzed by a statistical analysis tool in GraphPad Prism 9. For the aged K18-hACE2 mice challenged with the SARS-CoV-2 variants, the area under the curve (AUC) was calculated.

For the detection of anti-spike antibodies in mouse sera after low-dose immunization, SARS-CoV-2-specific IgG titers were determined by ELISA. In short, high-binding ELISA 96-well plates (Greiner Bio-One) were coated overnight at 4°C with 2 μg/mL of target protein in carbonate/bicarbonate coating buffer and then blocked for 2 h at RT. After incubation of samples on prepared plates, plates were washed with PBS and incubated with HRP-conjugated mouse IgG-specific antibodies for 1 h at RT. The OPD substrate was used to develop the reaction. The stop solution (1 M H_2_SO_4_) was added to stop the reaction. The absorbance of the samples was read using an optic reader at 490 nm, with 630 nm as the reference wavelength.

### Antibody avidity assay

Antibody avidity assays were performed as described by Welten et al. (2016) (27). In brief, 100 ng/well S1–S2 protein (SARS-CoV-2 Index or Omicron BA.1) were immobilized in carbonate buffer overnight at 4°C and plates were blocked with 2% MPBS-T for 1 h at RT. Plates were washed with H_2_O containing 0.05% Tween20 using EL405 S washer (BioTek). Murine sera with the highest IgG titers were selected and always three sera per group were pooled and diluted to a uniform IgG titer of 1:300 in 2% MPBS-T. After incubation at RT for 1 h, plates were washed as described above followed by incubation with NaSCN at RT in indicated dilutions. As a control, binding without NaSCN was included. After 15-min incubation, plates were washed immediately two times to ensure complete removal of NaSCN. Binding was detected using goat-anti mouse IgG antibody (A0168, Sigma) conjugated with HRP (1:42,000 dilution in 2% MPBS-T) at RT for 1 h and plates were washed as described above. Antibody binding was visualized using TMB substrate as described above, and the reaction was stopped after 15 min at RT using 0.5 M/1 N sulfuric acid. Absorbance was measured as described above, IgG binding was normalized to 100% in the absence of NaSCN, and the avidity was calculated as binding after treatment with indicated NaSCN dilutions on described antigens.

### Virus neutralization assay

The serum neutralization assay was performed as described before (17). Briefly, heat-inactivated sera were serially diluted and incubated with 100 PFU/100 μL of SARS-CoV-2 D614G for 1 h at RT. Thereupon, they were transferred to 96-well plates seeded with 2 × 10^4^ Vero-E6 cells per well on the day before. Cells were inoculated with virus and serum dilutions for 1 h at 37°C. After the inoculum removal, the cells were overlaid with 1.5% methylcellulose and incubated at 37°C and 5% CO_2_ for 3 days. The cells were fixed with 4% formaldehyde, followed by crystal violet staining and plaque counting. The serum-neutralizing titer that results in a 50% reduction of virus plaques (VNT_50_) was analyzed by GraphPad Prism 9 nonlinear regression analysis (inhibitor vs. response, variable slope, four parameters).

### Pseudo-virus neutralization assay

Pseudo-virus neutralization assays were performed as described in the previous publications (17, 74). In brief, 293T cells were transfected with pCG1 plasmids expressing different SARS-CoV-2 spike proteins, using calcium-phosphate. Twenty-four hours post-transfection, cells were infected with a replication-deficient reporter VSV-G (VSV ∗ ΔG-Fluc) at a multiplicity of infection (MOI) of 3 for 1 h at 37°C. Cells were washed once with PBS, and medium containing anti-VSV-G antibody (culture supernatant from L1-hybridoma cells) was added to neutralize residual input virus. The cell culture supernatant was harvested after 16 h, and cellular debris was removed by centrifugation at 2,000 × *g* for 5 min at 4°C. Aliquots were stored at −80°C until use. For pseudo-virus neutralization, serum samples and controls were heat-inactivated at 56°C for 30 min. Thawed samples and controls were stored at 4°C for no longer than 48 h, prior to use. In a 96-well microtiter plate, serum samples were twofold serially diluted in cell culture medium (DMEM, 5% FBS, 1% P/S, and 1% L-Glu) with a dilution range of 1:10 to 1:5,120. Pre-diluted samples were incubated with an equal volume of spike protein-bearing viral particles [approximately 200–500 fluorescence forming units (ffu)/well] at 37°C for 1 h. After incubation, the sample–virus mixture was transferred to VeroE6 cells at 100% confluence, which were seeded the day before. Cells were incubated at 37°C for 24 ± 2 h, while infected cells were visualized using an IncuCyte S3 (Sartorius) performing whole-well scans (4×) in phase contrast and green fluorescence settings. Automated segmentation and counting of fluorescent foci defined as green fluorescent protein (GFP)^+^ single cells was performed using the IncuCyte GUI software (versions 2019B Rev1 and 2021B). Raw data were plotted in GraphPad Prism version 9.0.2, and FRNT_50_ (focus reduction neutralization titer) was calculated with a variable slope, four-parameter regression analysis. Because regression analysis allows extrapolation of titers beyond the dilution range of the assay, we defined a lower limit of confidence (LLOC) as half of the lowest sample dilution tested restricting extrapolation to reasonable power. Values below that were below half of the LLOC were set to half of the LLOC.

### Bulk RNA extraction and sequencing

To perform RNA bulk sequencing, RNA was isolated from lung tissue and blood using Trizol reagent (Invitrogen) at a 1:3 ratio. Briefly, 3× Trizol LS was added to the homogenized organ sample or blood and vortexed thoroughly. After incubation, 1/5 of the volume of chloroform were added. The samples were vortexed again and incubated for 5 min at RT. Subsequently, tubes were centrifuged at 12,000 × *g* for 15 min at 4°C, and aqueous phase was transferred into a new tube and RNA was extracted with the RNA Clean and Concentrator kit (ZYMO Research).

Bulk RNA sequencing libraries were constructed using the NEBNext Ultra II Directional RNA Library Prep Kit (New England Biolabs) and sequenced on a high-throughput NextSeq 500 device. Reads were aligned to the *Mus musculus* genome (GRCm39 or mm39) using hisat2 (75) and gene expression was quantified using the package featureCounts from Rsubread (76). Analysis was done with DESeq2 (77). Differentially expressed genes were defined by an absolute fold change in mRNA abundance greater than 1.5 (log2 fold change of 0.58—using DESeq2 shrunken log2 fold changes) and an adjusted *p*-value of less than 0.05 (Benjamini–Hochberg corrected). Gene expression deconvolution was performed with the package GEDIT, using the reference matrix provided for blood analysis (78). Bulk RNA sequencing deconvolution was performed with the package granulator (79), using the cell-type annotations from scRNA-seq data of lung tissue homogenate (80) and blood (81) from SARS-CoV-2-infected Syrian and Roborovski hamsters as reference matrixes. The package dtangle was the method used for deconvolution analysis (82).

### Statistics

All information on statistical testing is provided in the respective figure legends. No formal testing of normal distribution was performed because we assumed normal distribution of all groups following biological consideration. Statistical analysis was calculated by GraphPad Prism 9.

## Data availability statement

All SARS-CoV-2 genome sequences are deposited in the GISAID or GenBank repositories, accession numbers: EPI_ISL_463008 (Delta), MW822592 (Beta), EPI_ISL_6959868 (Omicron BA.1). All other data of this study are available upon request.

## Ethics statement

The animal study was approved by the Lower Saxony State Office of Consumer Protection and Food Safety, license number: 33.19-42502-04-20/3580. The study was conducted in accordance with the local legislation and institutional requirements.

## Author contributions

KM: Conceptualization, Data curation, Formal analysis, Investigation, Methodology, Visualization, Writing – original draft, Writing – review & editing. HJ: Conceptualization, Data curation, Formal analysis, Investigation, Methodology, Writing – review & editing. YK: Conceptualization, Data curation, Formal analysis, Investigation, Methodology, Visualization, Writing – original draft, Writing – review & editing. LT: Data curation, Formal analysis, Investigation, Methodology, Visualization, Writing – review & editing. UK: Methodology, Writing – review & editing. KE: Methodology, Writing – review & editing. MC: Methodology, Writing – review & editing. MHo: Methodology, Writing – review & editing. FB: Methodology, Writing – review & editing. MR: Methodology, Writing – review & editing. MHu: Methodology, Resources, Validation, Writing – review & editing. MB: Methodology, Writing – review & editing. JM: Methodology, Writing – review & editing. MŠ: Methodology, Writing – review & editing. AK: Methodology, Writing – review & editing. SJ: Funding acquisition, Resources, Writing – review & editing. MW: Resources, Writing – review & editing. SC: Resources, Writing – review & editing. SP: Resources, Validation, Writing – review & editing. ML: Funding acquisition, Resources, Validation, Writing – review & editing. LČ-Š: Conceptualization, Funding acquisition, Project administration, Resources, Supervision, Validation, Writing – review & editing.
