# Supplementary material for: A single-dose MCMV-based vaccine elicits long-lasting immune protection in mice against distinct SARS-CoV-2 variants
Source: Front Immunol. 2024 Jul 25;15:1383086. doi: 10.3389/fimmu.2024.1383086 (PMC11306140; doi:10.3389/fimmu.2024.1383086)
Supplement: Supplementary file 1 [file DataSheet_1.docx]

**Supplementary Figures and Figure legends**

| 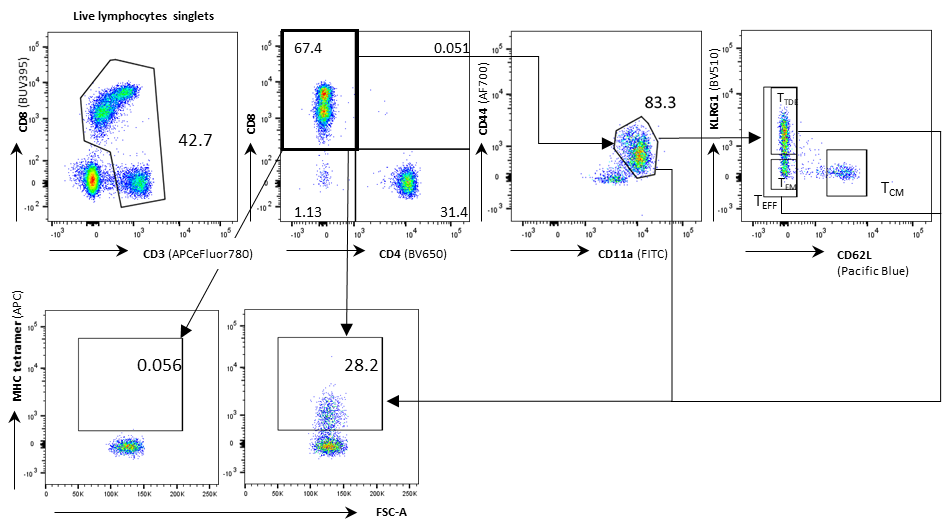 |
| --- |
| ***Supplementary Figure 1: Gating strategy for flow cytometric analyses.*** *Gating strategy for the analysis of VNFNFNGL-specific T cell subsets in mice. Lymphocytes and singlets were gated based on SSC and FSC; live cells were identified by staining with 7-AAD. CD8^+^ and CD4^-^ T cells were gated within the CD3^+^ population and progressively gated for primed cells (CD11^hi^ and CD44^hi^) and primed subsets, including T_EFF_ (CD62L^lo^), T_TDE_ (CD62L^lo^KLRG1^hi^), T_EM_ (CD62L^lo^KLRG1^lo^) or T_CM_ (CD62L^hi^KLRG1^lo^) subpopulations, which were further sub-gated for the antigen-specific subpopulations based on tetramer labeling.* |
| 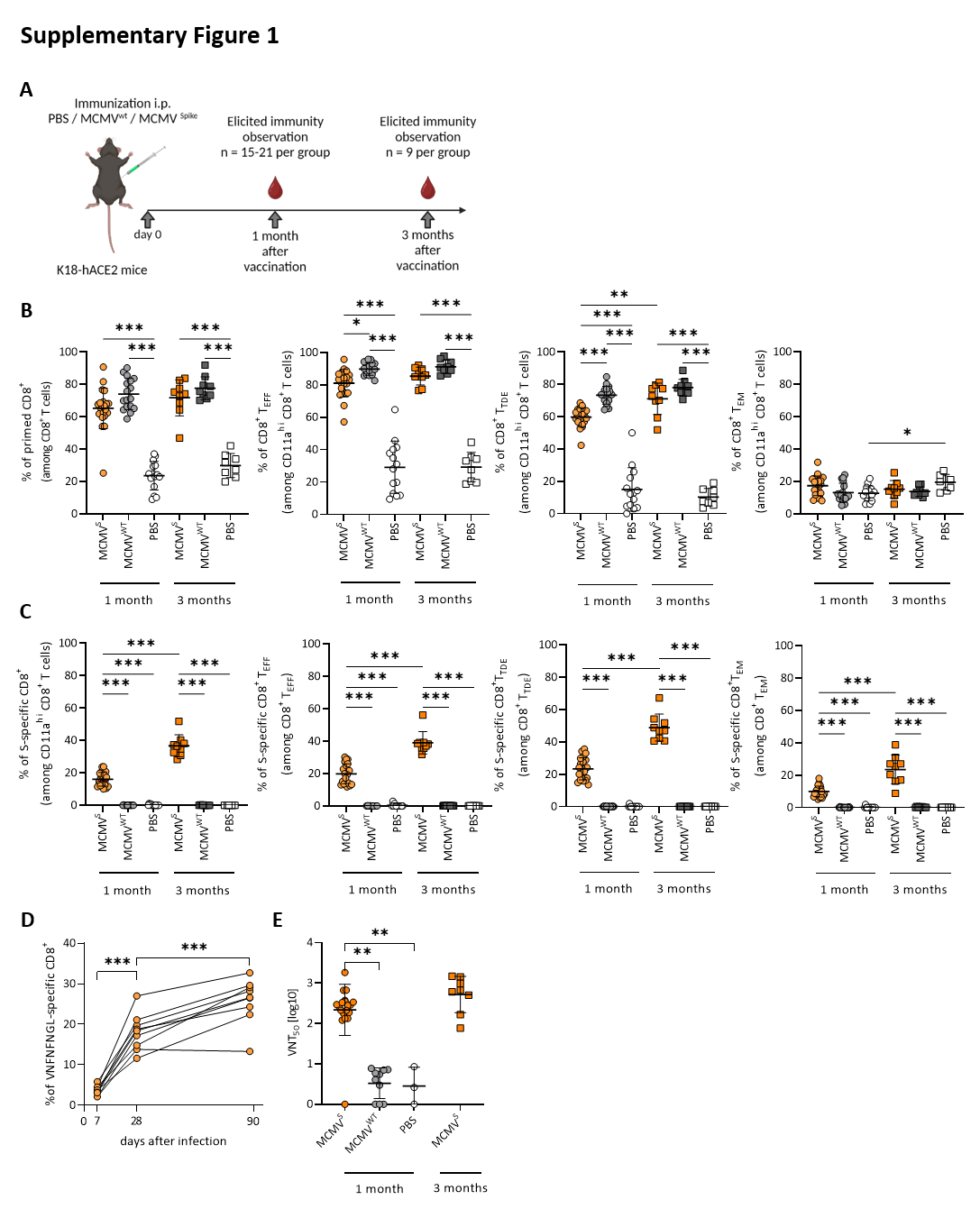 |
| ***Supplementary Figure 2: Protection against SARS-CoV-2 D614G-mediated disease elicited by a single dose of MCMV^S^-vaccine. (A)*** *Schematic representation of the experimental setup. Created with BioRender.com* ***(B)*** *CD3^+^CD8^+^CD4^-^ T cells from murine blood one month post-vaccination (PBS n=8, MCMV^WT^ n=9, MCMV^S^ n=9) or three months post-vaccination (PBS n=15, MCMV^WT^ n=17, MCMV^S^ n=21) were gated for the primed subpopulation (CD11^hi^CD44^hi^ - leftmost panel). Primed CD8^+^ T cells were then progressively gated into T_EFF_ (CD62L^lo^), T_TDE_ (CD62L^lo^KLRG1^hi^), or T_EM_ (CD62L^lo^KLRG1^lo^) subpopulations. Frequencies of cells in each subset as a fraction of the parental population are shown.* ***(C)*** *The percentages of SARS-CoV-2 Spike peptide (VNFNFNGL)-specific cells in each subset shown in (B) are shown.* ***(D)*** *VNFNFNGL-specific memory responses over time in murine blood from MCMV^S^-immunized mice (n=9) shown as the percentage of antigen-specific T cells within the total CD8^+^ compartment. Each line connects values from an individual mouse at the indicated time points.* ***(E)*** *Serum neutralization titers (VNT_50_) at one month (PBS n=3, MCMV^WT^ n=10, MCMV^S^ n=8) or three months (MCMV^S^ n=19) post-vaccination. Each symbol indicates an individual mouse. All data (except for (D) are shown as mean ± SD. Statistical assessments were performed using Brown-Forsythe and One-Way ANOVA and Dunnett T3 correction for multiple comparisons or Ordinary One-Way ANOVA with correction for multiple comparisons following Sidák if groups were uniformly negative (both two-tailed). (* p < 0.05, ** p <0.01, *** p<0.001).* |
| 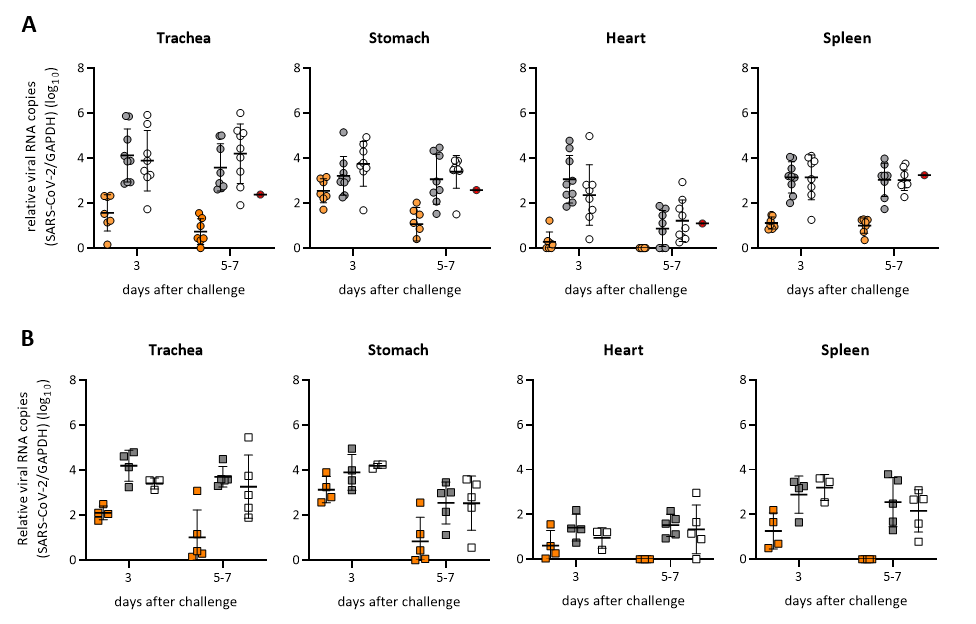 |
| ***Supplementary Figure 3: SARS-CoV-2 viral loads in immunized and control mice. (A-B)*** *Relative SARS‑CoV‑2 viral RNA loads in the representative organs (trachea, stomach, heart, spleen) of SARS-CoV-2 D614G-challenged mice at* ***(A)*** *six weeks* *(PBS n=8-9, MCMV^WT^ n=8-9, MCMV^S^ n=7) and* ***(B)*** *twelve weeks (PBS n=3-5, MCMV^WT^ n=4-5, MCMV^S^ n=4-5) post-immunization. One MCMV^S^-immunized animal is shown separately as crossed red symbol as this animal did not show any immunogenicity and is suspected to represent a technical vaccination-failure. Organs were harvested at the indicated time points of 3 and 5-7 days (restricted by animals reaching the humane endpoint before day seven) after SARS-CoV-2 D614G challenge. SARS-CoV-2 viral RNA was normalized to the housekeeping gene mGAPDH and log-transformed. Values below 1 were set to 1 for graphical representation (before transformation). All data are shown as mean ± SD. No statistical assessment was performed for any panel.* |

| 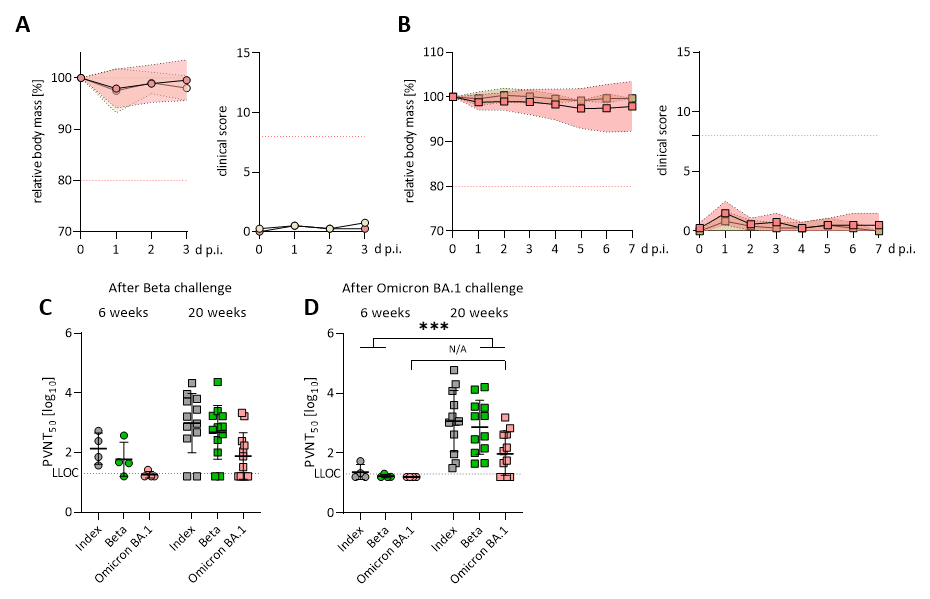 |
| --- |
| ***Supplementary Figure 4: Clinical presentation following SARS-CoV-2 Omicron BA.1-challenge and sustained neutralizing antibody responses against SARS-CoV-2 Beta and Omicron BA.1 upon MCMV^S^-immunization.*** ***(A)*** *Relative body mass (left) and clinical scoring (right) of MCMV^WT^-treated (n=4) or MCMV^S^-immunized (n=4) mice upon challenge with SARS-CoV-2 Omicron BA.1 at six weeks post-immunization.* ***(B)*** *Relative body mass (left) and clinical scoring (right) of MCMV^WT^-treated (n=12) or MCMV^S^-immunized (n=12) mice upon challenge with SARS-CoV-2 Omicron BA.1 at 20 weeks post-immunization. Mice were challenged with 2x10^3^ PFU of SARS-CoV-2 Omicron BA.1. (****C, D)*** *Post-challenge pseudo-virus serum neutralization titers (PVNT_50_) at six weeks (n=4; left) and 20 weeks (n=12; right) post-vaccination against SARS-CoV-2 Index, Beta and Omicron BA.1 variants. Titers were assessed three days after SARS-CoV-2* ***(C)*** *Beta and* ***(D)*** *Omicron BA.1 challenge. All data are shown as mean ± SD. Red-dotted lines* *indicate the maximal acceptable burden for animal experiments. The humane endpoint (>20% reduction in body mass or a clinical score of ≥8) was pre-defined by authorized animal trial permits. Black dotted lines indicate the lower limit of confidence (LLOC). Statistical significance for longitudinal assessments (A and B) was calculated with Greenhouse-Geisser corrected Two-Way ANOVA and Tukey post-hoc testing. Statistical assessments for panel (C and D), comparing all groups within one time-point were statistically assessed using Brown-Forsythe and One-Way ANOVA and Dunnett T3 correction for multiple comparisons (two-tailed) (*** p<0.001, N/A no comparison possible).* |

|  |
| --- |
| **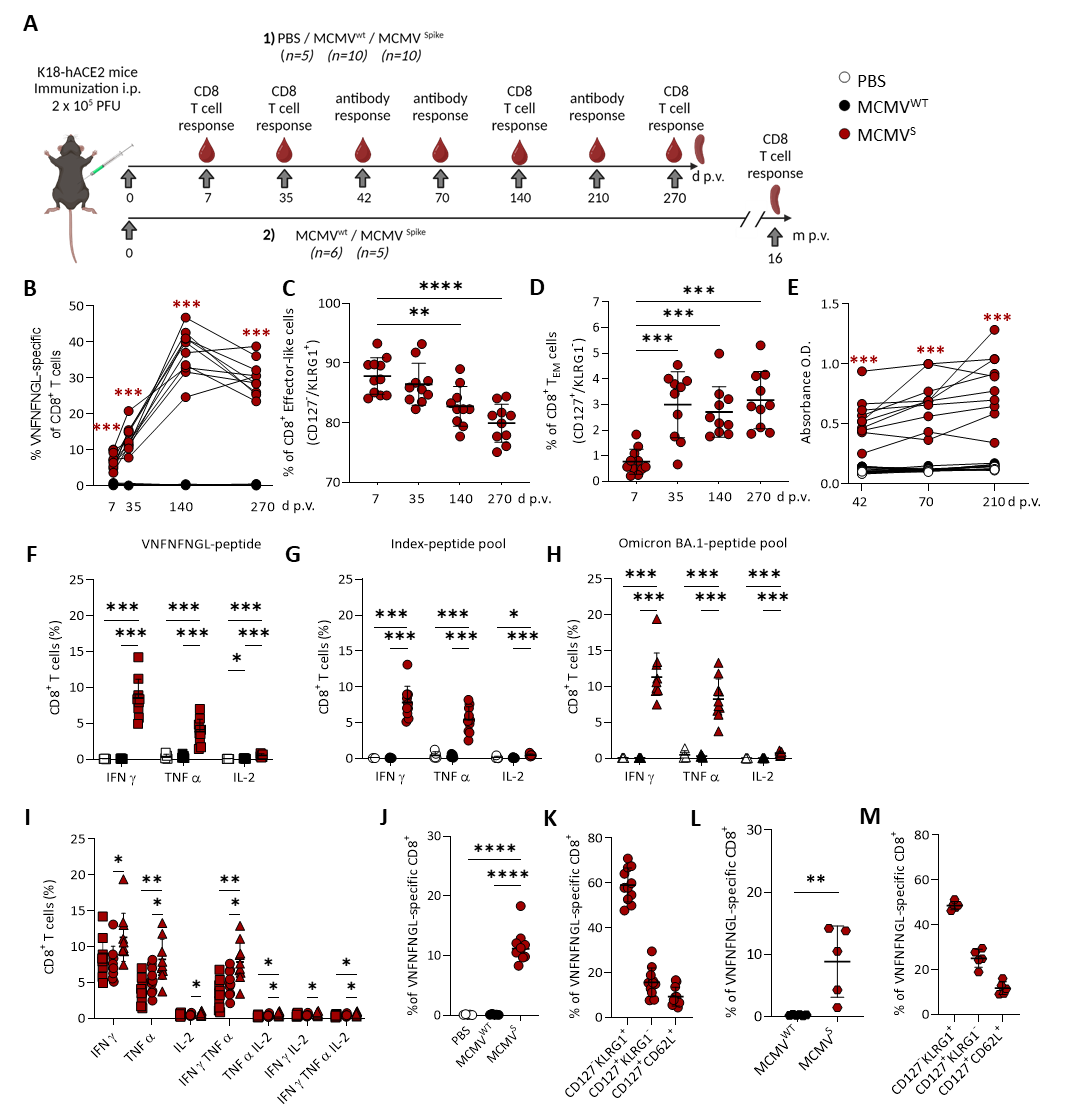**  ***Supplementary Figure 5: Low-dose immunization with MCMV^S^ elicits long-lasting immunogenicity in aged mice. (A)*** *Schematic representation of the experimental setup. Mice were treated with PBS or immunized with*  *2x10^5^ PFU of either MCMV^S^, MCMV^WT^  in all settings.* *Created with BioRender.com.* ***(B-J)*** *CD8 T cell response and SARS-CoV-2 antibody response in aged K18-hACE2 mice were measured in the blood and followed up to 270 d p.v. after immunization with a low dose (2x10^5^ PFU) of MCMV^S^ (PBS n=5, MCMV^WT^ n=10, MCMV^S^ n=10).* ***(B)*** *Longitudinal frequency of VNFNFNGL-specific memory responses over time in murine blood from MCMV^S^-immunized mice (n=9) shown as the percentage of antigen-specific T cells within the total CD8+ compartment. Each line connects values from an individual mouse at the indicated time points.* ***(C-D)*** *Primed CD8 T cells of MCMV^S^-immunized mice were progressively gated into antigen-specific effector-like cells: CD8^+^/CD44^+^/Tet^+^/CD127^-^/KLRG1^+^* ***(C)*** *and effector-memory cells (T_EM_): CD8^+^/CD44^+^/Tet^+^/CD127^+^/KLRG1* ***(D). (E)*** *Longitudinal anti-Spike IgG response in the sera of MCMV^S^ immunized K18-hACE2 mice were determined by ELISA and visualized by the Absorbance O.D..* ***(F-H)*** *Measurement of the immune responses of mice treated with PBS (white) or immunized with either MCMV^WT^  (black) or MCMV^S^(red) using the VNFNFNGL-peptide* ***(F)****, a peptide pool of overlapping peptides covering the S-protein of Index-Sars-CoV-2* ***(G)*** *or Omicron BA.1.* ***(H)****.* ***(I)*** *IFN-γ, TNF-α and IL-2 production by CD8 T cells was stimulated using the VNFNFNGL-peptide, a peptide pool of overlapping peptides covering the S-protein of Index-Sars-CoV-2 or Omicron BA.1. in MCMV^S^ immunized mice****. (J-K)*** *Frequency of SARS-CoV-2 Spike peptide (VNFNFNGL)-specific CD8 T cells in murine spleens* *after 270 d p.i..* ***(K)*** *Primed CD8 T cells of MCMV^S^-immunized mice were progressively gated into antigen-specific effector-memory cells (T_EM_): CD8^+^/CD44^+^/Tet^+^/CD127^+^/KLRG1^-^, effector-like cells: CD8^+^/CD44^+^/Tet^+^/CD127^-^/KLRG1^+^ or central memory cells (T_CM_): CD8^+^/CD44^+^/Tet^+^/CD127^+^/CD62L^+^ after 270 d p.v..* ***(L-M)*** *Immune response in aged K18-hACE2 mice at 16 months after immunization of MCMV^S^.* ***(L)*** *Frequency of SARS-CoV-2 Spike peptide (VNFNFNGL)-specific CD8 T cells in murine spleens (MCMV^WT^ n=6, MCMV^S^ n=5).* ***(M)*** *Primed CD8 T cells of MCMV^S^-immunized mice were progressively gated into antigen-specific effector-memory cells (T_EM_): CD8^+^/CD44^+^/Tet^+^/CD127^+^/KLRG1^-^, effector-like cells: CD8^+^/CD44^+^/Tet^+^/CD127^-^/KLRG1^+^ or central memory cells (T_CM_): CD8^+^/CD44^+^/Tet^+^/CD127^+^/CD62L^+^.*  *All data are shown as mean ± SD. Statistical assessment of panel (B and E) was calculated with Greenhouse-Geisser corrected Two-Way ANOVA and Tukey post-hoc testing. All other comparisons (C,D and F-M) were statistically assessed using Brown-Forsythe and One-Way ANOVA and Dunnett T3 correction for multiple comparisons (two-tailed). (* p<0.05, ** p <0.01, *** p < 0.001).* |

| 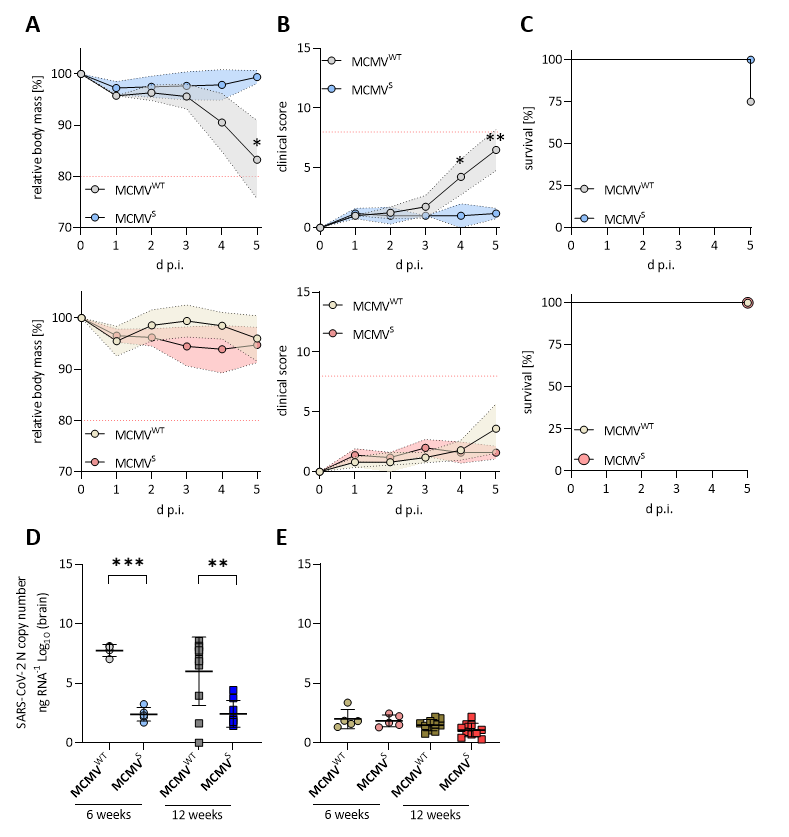 |
| --- |
| ***Supplementary Figure 6:*** ***Aged MCMV^S^-immunized mice show long-lasting protection against heterologous SARS-CoV-2 infection. (A)*** *Relative body mass,* ***(B)*** *clinical scoring, and* ***(C)*** *survival of mice that were challenged with the SARS-CoV-2 Delta variant (top) or Omicron BA.1 variant (bottom) at six weeks post-immunization with MCMV^WT^ (n=4-5) or MCMV^S^ (n=5).* *Mice were challenged with 2x10^3^ PFU of SARS-CoV-2 Delta or Omicron BA.1.* ***(D,E)*** *Viral loads as SARS-CoV-2 N gene copy numbers per ng RNA in murine brains at day five following SARS-CoV-2 Delta* ***(D)*** *or Omicron BA.1* ***(E)*** *infection at six weeks (MCMV^WT^ n=4-5, MCMV^S^ n=5) or twelve weeks (MCMV^WT^ n=10-11, MCMV^S^ n=8-10). All data (except for C) are shown as mean ± SD. Red-dotted lines indicate the maximal acceptable burden for animal experiments. The humane endpoint (>20% reduction in body mass (A) or a clinical score of ≥8 (B)) was pre-defined by authorized animal trial permits. For survival analyses, a log-rank (Mantel-cox) test was used to assess statistical significance. Statistical significance for longitudinal assessments (A and B) was calculated with Greenhouse-Geisser corrected Two-Way ANOVA and Tukey post-hoc testing. All other comparisons (D and E) were statistically assessed using Brown-Forsythe and One-Way ANOVA and Dunnett T3 correction for multiple comparisons (two-tailed). (* p<0.05, ** p <0.01, *** p < 0.001).* |


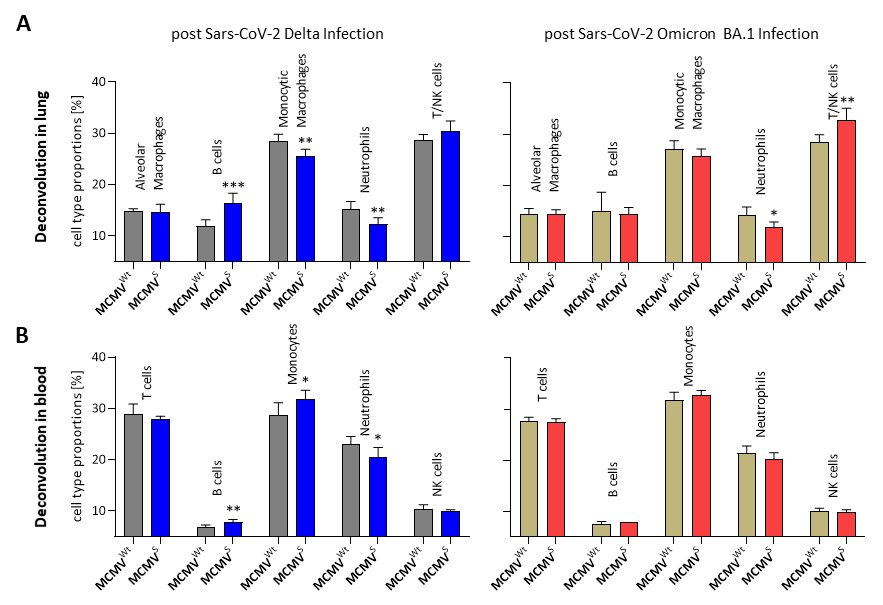


***Supplementary Figure 7:*** ***Cell-type deconvolution of bulk RNA sequencing data from lung and blood.*** *Cell-type deconvolution of bulk RNA sequencing data from lung and blood. Cell-type proportion predicted by deconvolution of bulk RNA-sequencing data performed with the package granulator for cell type prediction, based on lung tissue and blood from SARS-CoV-2-infected hamsters, as reference matrix. Shown are fractions of the cell type selected of the total count.* ***(A-B)*** *Deconvolution in lung* ***(A)*** *tissue and blood* ***(B)*** *of mice challenged with SARS-CoV-2 Delta (left) or Omicron BA.1 (right) at twelve weeks post-immunization (MCMV^WT^ n=11, MCMV^S^ n=9). The cell-type proportion predicted for* ***(A)*** *Alveolar Macrophages, B cells, Monocytic macrophages, Neutrophils and both T cell and NK cells (T/NK cells) and* ***(B)*** *T cells, B cells, Monocytes, Neutrophils and NK cells. Add data are shown as mean ± SD. Statistical significance was calculated using Brown-Forsythe and One-Way ANOVA and Dunnett T3 correction for multiple comparisons (two-tailed). (* p < 0.05, ** p <0.01, *** p < 0.001).*
